# Evaluating Drug–Drug Interactions by Constructing Advanced Machine Learning Models of ABC Transporter Inhibitors

**DOI:** 10.1021/acs.jcim.6c02847

**Published:** 2026-10-26

**Authors:** Sean R. Carey, Xia Wen, Nada J. Daood, Elena Chung, Jacqueline B. Tiley, Lauren M. Aleksunes, Hao Zhu

**Affiliations:** † Center for Biomedical Informatics and Genomics, 5783 Tulane University School of Medicine, New Orleans, Louisiana 70112, United States; ‡ Department of Chemistry and Biochemistry, Rowan University, Glassboro, New Jersey 08028, United States; § Department of Pharmacology and Toxicology, 43337 Rutgers University, Piscataway, New Jersey 08854, United States; ∥ Division of Pharmacotherapy and Experimental Therapeutics, UNC Eshelman School of Pharmacy, University of North Carolina at Chapel Hill, Chapel Hill, North Carolina 27599, United States

## Abstract

ATP-binding cassette (ABC) transporters play a critical role in transmembrane transport, and the inhibition of ABC transporter-mediated efflux can lead to unintended drug–drug interactions (DDIs). The identification of new drug candidates as perpetrators of transporter-mediated DDIs requires synthesis of significant drug products and costly experimental evaluation. Alternatively, computational approaches can rapidly and accurately screen the DDI liability of new drug candidates *in silico*. Here, we developed a novel DDI risk assessment protocol incorporating classic and graph-based quantitative structure–activity relationship (QSAR) models. These models were trained on publicly available, large-scale, structurally diverse *in vitro* chemical inhibition data for P-gp, BCRP, and MRP1. The resultant models yielded promising R^2^ scores (0.58 to 0.82). External predictions successfully identified inhibitors, verified by experimental validation or literature review, and detected drugs with the potential to perpetrate clinically relevant DDIs. In summary, we developed a large, diverse database of ABC transporter inhibitors and present an applicable alternative protocol that incorporates model predictions to evaluate transporter-mediated DDIs for new drug candidates to prevent potentially harmful clinical outcomes.

## Graphical abstract

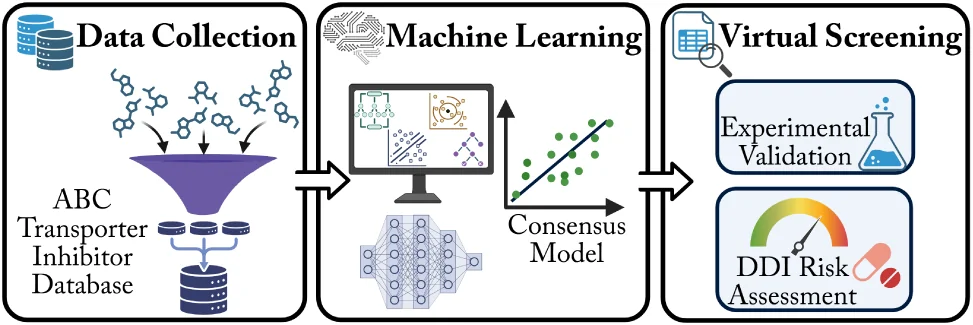

## Introduction

ATP-binding cassette (ABC) transporters are widely expressed cell membrane efflux transporters that regulate transcellular transfer of xenobiotics and endogenous substances, often serving as a protective barrier against potentially hazardous chemicals.^1–5^ Consequently, when ABC transporter efflux is influenced by intrinsic (e.g., genetics, age, sex, or disease) or extrinsic (e.g., drug interactions or environmental exposure) factors, potential adverse or unintended outcomes can arise.^1^ Extensive research efforts have explored ABC transporter structures, functions, and tissue expression across the human body. Multidrug resistance P-glycoprotein (P-gp; also known as MDR1), breast cancer resistance protein (BCRP), and multidrug resistance-associated protein 1 (MRP1), three of the most well-characterized members of this protein superfamily, are predominantly expressed in the intestine, kidney, liver, and placenta.^1,3,4,6–8^ P-gp, BCRP, and MRPs located at the brain, retina, and reproductive tissues act as barriers for drugs and environmental contaminants.^9^ Furthermore, intestinal, hepatic, and renal P-gp and BCRP play an important role in regulating drug pharmacokinetics.

As a result, regulatory agencies require screening of new chemical entities as potential ABC transporter inhibitors to avoid transporter-mediated drug–drug interactions (DDIs). For instance, the current United States Food and Drug Administration (FDA) industry guidelines provide a stepwise approach to evaluate new drugs as potential inhibitors of various transporters and determine the necessity of clinical DDI studies.^10^ Under these guidelines, *in vitro* studies, involving numerous testing procedures or complex experimental systems, are required. Various *in vitro* systems for the evaluation of chemical-transporter interactions are highlighted, including cell-based and membrane vesicle assays. Cell-based systems, such as human colon adenocarcinoma cells, human embryonic kidney 293 (HEK293) cells, and Madin–Darby canine kidney cells, have been extensively utilized to determine inhibition of ABC transporters *in vitro*.^11,12^ Membrane vesicular transport assays have also been used to evaluate numerous efflux transporters.^13–15^ These relatively low-cost approaches allow for effective high-throughput testing of investigational chemicals as inhibitors. Alternatively, three-dimensional (3D) systems, including cell culture models and microphysiological systems (MPS), have been used to evaluate biological mechanisms involved in drug disposition.^16^ Here, several applications of the 3D systems methodologies are described, including a duodenum intestine-chip from organoid-derived cells to study drug transport and DDIs^17^ and disease modeling of the blood–brain barrier using MPS and induced pluripotent stem cells.^18^ While these *in vitro* approaches can directly identify ABC transporter chemical interactions and provide key insights into relevant biological processes, several limitations persist. Currently, these approaches lack a universal, standardized protocol, resulting in interlaboratory variance^10,19^ and challenges for broad *in vitro*-to-*in vivo* extrapolation.^1,20–22^ Furthermore, complex systems which can better capture biological processes have limited high-throughput capabilities and reproducibility.^23^ Finally, the synthesis and screening of sufficient quantities of investigational drug candidates in order to meet regulatory requirements for assessing DDI risk is both labor- and resource-intensive.

Following recent advancements in machine learning (ML) and deep learning (DL) approaches and the availability of empirical testing data from public resources,^24–28^ computational models have shown promise in supporting these experimental efforts by detecting potential liabilities of drug candidates in the early stages of drug discovery and development. In this way, computational models can alleviate the burden of chemical synthesis and testing during initial screenings, especially when a significant number of chemicals are being investigated. Historically, computational models assessing ABC transporter inhibition span decades of research and publications.^29–49^ Computational modeling strategies, such as quantitative structure–activity relationship (QSAR) and physiologically based pharmacokinetic (PBPK) models, have been applied to study the inhibition of ABC transporters. Likewise, previous QSAR studies interrogating potential ABC transporter inhibitors have employed a variety of ML algorithms, data sets, and chemical descriptors.^50^ These models aid drug discovery by removing unsuitable candidates prior to further drug development efforts. In addition, PBPK modeling has been extensively used to study transporter-related processes, such as drug absorption, distribution, and clearance, and is commonly used to support new drug applications and other regulatory agency submissions.^1,19,21,51^ However, while these previous computational studies have yielded encouraging results, they have been limited by relatively small training data sets or utilized proprietary data that are not publicly available to the broader scientific community.^48,49,52^ Moreover, models which are currently available online or in the literature often provide binary inhibition classifications rather than potency predictions.^53,54^ Finally, few efforts have attempted to directly apply transporter QSAR models for the broader evaluation of DDI liability.

To address the above challenges, we developed a QSAR modeling workflow aimed at identifying drugs as inhibitors capable of perpetrating clinically relevant ABC transporter-mediated DDIs. Despite the clinical significance of numerous membrane transporters in the ABC transporter family, many lack sufficient data to construct reliable IC_50_ models. As such, P-gp, BCRP, and MRP1, the ABC transporters with the most abundant publicly available data, were selected for this study. Bioactivity data related to the inhibition of P-gp, BCRP, and MRP1 were compiled, yielding large, diverse modeling sets containing approximately 2,200 unique compounds. QSAR models were built on classical and graphical molecular representations, and model outputs were averaged to obtain consensus models used for external predictions on two public data sets: DrugBank (https://go.drugbank.com/, accessed August 2025) and Inxight Drugs (https://drugs.ncats.io/, accessed August 2025). External DrugBank predictions effectively identified previously reported P-gp and BCRP inhibitors, while those on the Inxight Drugs data set successfully classified known perpetrators of P-gp- or BCRP-mediated DDIs. Our results suggest that the proposed computational strategy can be integrated into the drug discovery process to detect potential ABC transporter inhibitors and evaluate novel inhibitors responsible for clinically significant DDIs.

## Methods

### Data Collection and Curation

The transporter inhibition data used in this study were obtained from the online ChEMBL database (https://www.ebi.ac.uk/chembl/, accessed August 2025).^55^ Data for the selected transporters were programmatically collected in Python v.3.9.16 using the chembl_webresource_client package provided by ChEMBL. To increase data set sizes, transporter inhibition data were also collected from the Papyrus data set (version 05.5) – a publicly available benchmark data set containing high-quality data for bioactivity prediction.^56^ A standardized data curation protocol was implemented for the originally collected data sets. The standard, harmonized pChEMBL value created by ChEMBL and used by Papyrus, which takes concentration-based activities (e.g., IC_50_) at molar units and normalizes them on a negative log scale (e.g., pIC_50_), was used in this study as the endpoint for transporter inhibition. To ensure the quality of the modeling sets, the following curation protocol was adopted: (1) entries with data validity comments (i.e., “Outside typical range”) were removed; (2) only *in vitro* IC_50_ records were kept to avoid conflicting results generated from different endpoints (e.g., K_i_ and EC_50_); (3) records reporting ranges of activities were removed. These criteria aimed to address data reliability concerns related to variability across different protocols and measured endpoints which are present in heterogeneous data resources.

Chemical structures were represented by SMILES strings that were standardized using the PubChem Identifier Exchange Service (https://pubchem.ncbi.nlm.nih.gov/idexchange/, accessed August 2025), which converted the provided SMILES strings to standardized canonical SMILES. Molecular representations were generated in Python using the open-source RDKit (v.2024.03.5) toolkit, where mixtures were cleaned, charges were neutralized, and the standardized molecules were compressed into 2D representations, removing stereochemistry and chirality. 2D molecular representations were used to limit computational burden during feature generation by reducing feature complexity and have been shown to achieve satisfactory model performance.^25,28,57,58^ The CASE Ultra DataKurator (v.1.9.0.8) was used to detect and merge duplicate entries within the data sets by averaging associated bioactivities (MultiCASE Inc., Mayfield Heights, OH). An in-house data curation program was utilized to ensure clean molecular representations. The data curation process resulted in three data sets for model development purposes (Table S1, Figure S1).

To validate model performance and investigate the application of the developed models toward DDI evaluation, two external prediction sets were utilized in this study. The DrugBank database contains over 12,000 investigational, experimental, approved, and withdrawn drugs (https://go.drugbank.com/, v5.1.13, accessed August 2025).^59^ From the National Center for Advancing Translational Sciences, the Inxight Drugs Fast Response Database (FRDB) provides drug-related pharmacokinetic, adverse event, and DDI data for approximately 4,500 drug molecules at various stages of FDA approval or clinical trials (https://drugs.ncats.io/, v2024-12-30, accessed August 2025).^60^ The FRDB contains pharmacokinetic data for approximately 3,000 drugs. These data were curated to only retain entries reporting maximum plasma concentration (*C*_max_) values in humans. If there were duplicate entries for a drug, then the average of the minimum and maximum reported *C*_max_ values was used. For both external data sets, the same data curation process was performed as described above.

### Chemical Descriptors and Molecular Graphical Representations

Four chemical descriptor sets were calculated as QSAR model inputs: Extended-Connectivity Fingerprints (ECFP6), Functional-Connectivity Fingerprints (FCFP6), Molecular ACCess System (MACCS) keys, and RDKit physicochemical descriptors (RDKit). ECFP6 and FCFP6 are Morgan fingerprints that, using a bond radius of 3, calculate 1024-bit binary vectors that encode substructure-based atom or functional group information, respectively.^61^ MACCS keys consist of 166 predefined binary fingerprints representing the presence or absence of molecular fragments and substructural data. RDKit descriptors encompass 124 physicochemical and topological features. These four descriptor sets were generated using the RDKit toolkit. Besides chemical descriptors, graphical representations were created as DL modeling inputs using the ConvMolFeaturizer class provided by DeepChem (v.2.7.2.dev), an open-source Python library which provides ML and DL tools for drug discovery and other applications in life sciences.^62^ The ConvMolFeaturizer class converts chemical structures into graphs from their SMILES strings, where nodes represent atoms and edges represent bonds, generating descriptor vectors for each atom to directly input molecules as graphical inputs for convolutional neural network training.^63^

### QSAR Model Development

Having shown strong predictive performance in previous studies,^57,58,64,65^ four ML algorithms, random forest (RF), support vector machine (SVM), extreme gradient boosting (XGB), and *k*-nearest neighbors (*k*NN), were selected for this study. Although there are other ML algorithms available for model development, we focus on the use of consensus predictions, which are not sensitive to the inclusion of additional modeling methods.^66–69^ RF, SVM, and *k*NN regressors were implemented using the scikit-learn (v1.3.0) RandomForestRegressor, SVR, and KNeighborsRegressor classes, respectively. XGB was implemented using the xgboost (v2.0.3) XGBRegressor class. RF takes the average of predictions from an ensemble of decision trees built from randomized subsets of the training data.^70^ SVM uses an optimization framework which allows prediction errors within a defined tolerance range to find the best-fitting function and minimize model complexity.^71,72^ *k*NN predicts the activity of a compound based on the known activities of its *k* nearest training set compounds in a defined chemical space.^73^ XGB is an ensemble, gradient boosting algorithm that uses a sequential decision tree optimization process, where each subsequent decision tree corrects the errors of the previous decision tree.^74^ Each classical QSAR model was optimized using the scikit-learn grid-search algorithm, which performs hyperparameter tuning to identify the model variant with the highest-performing combination of input parameters for each ML algorithm (i.e., number of decision trees for RF, number of training set chemicals to consider as neighbors for *k*NN).

The GraphConvModel class in the DeepChem library was used to develop a graph convolutional network (GCN) model. GraphConvModel takes each molecular graph and passes it through a series of graph convolutional layers, which aggregates information from neighboring atoms to update each atom’s feature vector. A graph pooling layer combines all atom feature vectors to produce the complete molecular representation as a single vector. This vector is then passed to the fully connected dense layer, which outputs a single regression value.

Each modeling set was randomly split into a training set (85%) and test set (15%). All models underwent 5-fold cross-validation (5FCV) as described in our previous studies.^26–28,58,64^ Briefly, each training set was randomly divided into five equal subsets. During 5FCV, the model is trained on four of the five training subsets, making predictions on the final subset. This process is repeated until each subset is left out for prediction once. Model performance was assessed using coefficient of determination (R^2^), which evaluates the correlation between predicted and experimental activities. The models with the best 5FCV performance were then used to predict the test sets that were held out of model training.

### Applicability Domain

A chemical similarity-based applicability domain (AD) was applied to evaluate the impact of chemical diversity on model performance. Tanimoto similarity was used to determine chemical similarity between training and test set compounds, where a score of 1 represents identical compounds (according to the set of chemical features used), and a score of 0 represents no shared features between two compounds.^75^ Test compounds with no training compounds within a given similarity threshold were considered out of domain. After calculating the Tanimoto similarity score between training and test compounds and removing dissimilar compounds, model performance trends were analyzed across various thresholds.

### DDI Risk Assessment and Evaluation

The proposed DDI risk evaluation scoring system includes ratio- and probability-based scores to assess DDI risk. Based on FDA guidelines,^10^ eq 1 was used to evaluate drugs potentially acting as inhibitors of P-gp or BCRP following intestinal absorption, where DDI Ratio is the ratio of a drug’s *C*_max_ to its model predicted IC_50_.

1 $$\mathrm{DDI}\;\mathrm{Ratio}=\frac{C_{\max}}{{\mathrm{IC}}_{50}}$$

The probability-based score incorporates QSAR inhibitor probability predictions from our recent publication.^57^ Each individual score is given a value from 0 to 4 based on the criteria provided in Table S2. Briefly, FDA guidelines recommend that a chemical with a DDI Ratio value greater than 0.02 should be considered as a potential DDI perpetrator and requires additional DDI liability testing. Thus, 0.02 was selected as a cutoff value for ratio scores. To represent lower and higher risk DDI perpetrators, additional ratio cutoffs at 0.005, 0.05, and 0.1 were also included. Given the scale of the probability scores ranging from 0 to 1, the thresholds were equally split into five groups to match the ratio score splits. The final dual-criteria Total Score takes the sum of the ratio score and probability score, resulting in scores ranging from 0 to 8. The labels “Low Risk”, “Moderate Risk”, and “High Risk” are given to drugs with Total Scores of 0–4, 5–6, and 7–8, respectively.

### Experimental Validation

#### Inhibition of BCRP or P-gp Activity in Overexpressing Cells

*ABCB1* cDNA (Origene, Rockville, MD) was transfected into Flp-In 293 cells (ThermoFisher Scientific, Carlsbad, CA) containing an integrated FRT site. In detail, Flp-In 293 cells were seeded in 6-well plates (7 × 10^5^/well) overnight. Opti-MEM I reduced serum media (ThermoFisher Scientific) was used for the transfection of pcDNA5/FRT/MDR1 or pcDNA5/FRT (empty vector, EV), pOG44 and lipofectamine LTX (ThermoFisher Scientific). Stable cell lines were selected by using hygromycin B (200 μg/mL) as a selection marker. Colonies were isolated and screened for P-gp expression by Western blot analysis (Figure S2). Flp-In 293 EV and P-gp cells (ThermoFisher Scientific) were grown at 37 °C with 5% CO_2_ in Dulbecco’s modified eagle medium (ThermoFisher Scientific) supplemented with 10% of fetal bovine serum, 1% of glutamine (200 mM) and zeocin (100 μg/mL). HEK293 cells stably expressing an empty vector (EV, pcDNA 3.1) and the human BCRP/*ABCG2* were kindly provided by Dr. Robert Robey from the National Cancer Institute, National Institutes of Health in Bethesda, MD (Morisaki et al., 2005, Bircsak et al., 2016). Cells were cultured in Dulbecco’s modified Eagle medium (ThermoFisher Scientific) supplemented with 10% fetal bovine serum (Atlanta Biologicals, Norcross, GA), 1% penicillin-streptomycin, and 0.2 mg/mL Geneticin (ThermoFisher Scientific). BCRP protein expression was verified by Western blot analysis (Figure S2).

The EV, P-gp or BCRP cells (2 × 10^4^/well) were seeded on poly-d-lysine coated black 96-well plates (Corning, Corning, NY) overnight and treated with tested chemicals (0–100 μM or 0–10 μM) or positive inhibitors (2 μM of PSC 833 or Ko143 used for P-gp or BCRP, respectively) including P-gp substrate Rhodamine 123 (Cayman Chemical, Ann Arbor, MI) and Hoechst 33342 (Sigma St. Louis, MO, 0.5 μM) or BCRP substrate Hoechst 33342 (10 μM) in Hanks balanced salt solution for 30 min (*uptake period*). After washing cells with PBS, the tested chemicals in Hanks balanced salt solution were added for 60 min (*efflux period*). The fluorescence intensity of Rhodamine 123 (for P-gp) or Hoechst 33342 (for BCRP) normalized to cell numbers (counted by Hoechst 33342) was determined using Cytation 5 Imaging multimode reader (Agilent Technologies, Santa Clara, CA, YFP filter cube with excitation 500/24 nm, emission 542/27 nm for Rhodamine 123; DAPI filter cube with excitation 377/50 nm, emission 447/60 nm for Hoechst 33342).

#### Inhibition of BCRP in Inverted Plasma Membrane Vesicles

Compounds exhibiting BCRP inhibition with IC_50_ values greater than 10 μM were selected for subsequent assessment of their inhibitory activity in inverted membrane vesicles from cells expressing human BCRP. Specifically, 20 μg of membrane vesicles were preincubated with the BCRP substrate lucifer yellow (20 μM) and vehicle control, each test compounds (50 or 100 μM), or the positive inhibitor Ko143 (2 μM), in reaction buffer (50 mM MOPS–Tris, 70 mM KCl, 7.5 mM MgCl_2_) at 37 °C for 15 min. The mixture was then incubated with 10 mM MgATP for 2 min. Reactions were stopped by adding ice-cold buffer (40 mM MOPS–Tris, 70 mM KCl) and transferred to a 96-well Multiscreen filter plate (Millipore, Billerica, MA). After washing, vacuum filtration, and solubilization with 50% methanol, lucifer yellow fluorescence was measured at an excitation wavelength of 430 nm and an emission wavelength of 538 nm. The concentration of lucifer yellow was expressed as pmol/μg of membrane vesicle protein. The inhibitory effects of the test chemicals were presented as a percentage of the vehicle control. Of note, the complementary use of both assays provides a more comprehensive evaluation of inhibitory activity by accounting for both cellular and membrane level effects. However, the IC_50_ values obtained from overexpressing cells and inverted membrane vesicles are not directly comparable, as they are influenced by assay-specific factors.

The percentage of inhibition was calculated using the following equation^10,76–78^ and the nonlinear regression with best-fit curve analysis (GraphPad Prism 10.0 Software, San Diego, CA) was used to determine the half-maximal inhibitory concentration (IC_50_).

2 $$\mathrm{Percent}\;\mathrm{Inhibition}=\left(V_{\mathrm{inh}}-V_{\mathrm{control}}\right)/\left(V_{\mathrm{positive}\;\mathrm{inhibitor}}-V_{\mathrm{control}}\right)\times 100$$

In eq 2, V_inh_ is the fluorescence intensity of Rhodamine 123 or Hoechst 33342 in the presence of the tested chemicals; V_control_ is the fluorescence intensity of Rhodamine 123 or Hoechst 33342 in the absence of the tested chemicals; V_positive inhibitor_ is the fluorescence intensity of Rhodamine 123 or Hoechst 33342 in the presence of a well-known inhibitor (PSC 833 for P-gp or Ko143 for BCRP).

## Results and Discussion

### Modeling Workflow

The modeling workflow is shown in Figure 1. Bioactivity data for P-gp, BCRP, and MRP1 were gathered from ChEMBL and Papyrus. Curation of these data yielded three data sets (Table S1) for model development. Classic QSAR models (built on established ML algorithms and descriptors) and one GCN model were developed for each transporter data set. External predictions on the DrugBank and Inxight Drugs data sets were used to explore the ability of the resultant models to detect inhibitors and DDI-perpetrating drugs. New chemicals selected based on model predictions for P-gp and BCRP were experimentally validated using overexpressing cells and fluorescent substrate accumulation assays.

**Figure 1:**
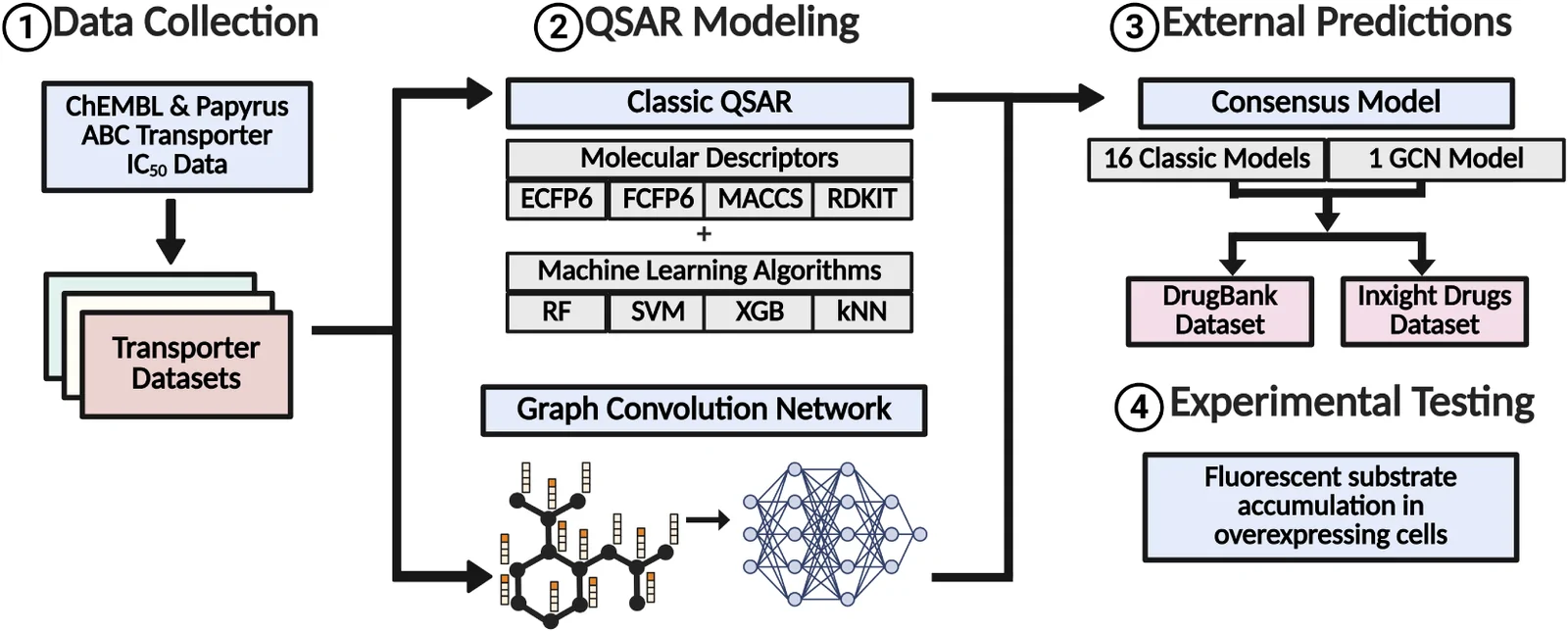
Overview of modeling workflow. *Created with Biorender.com*.

### Data Collection and Curation

Bioactivity records were obtained from ChEMBL and Papyrus using the respective target identifiers for P-gp (CHEMBL4302; P08183_WT), BCRP (CHEMBL5393; Q9UNQ0_WT), and MRP1 (CHEMBL3004; P33527_WT). In total, approximately 24,000 records were retrieved. A comparison of the ChEMBL and Papyrus records found no significant discrepancies across bioactivity distributions and ranges. After initial data cleaning and merging, the P-gp, BCRP, and MRP1 data sets contained 2,343, 1,588, and 499 compounds, respectively. For compounds with duplicate entries, we investigated two duplicate handling approaches: (1) taking the mean bioactivity; (2) taking the most potent reported bioactivity. It was found that QSAR models trained on mean activities showed stronger performance than those using the most potent activities (data not shown). Furthermore, it is possible that the highest reported bioactivities may include experimental outliers resulting from experimental errors, whereas the mean activity duplicate handling strategy aimed to retain more representative activities for chemicals with abundant IC_50_ records. For example, 89 IC_50_ records exist for verapamil (CHEMBL6966) against P-gp, with a mean pIC_50_ of 5.99 ± 1.2 and most potent recorded pIC_50_ of 8.53. Given the heterogeneity of the transporter data sets (i.e., different cell lines and experimental protocols), an additional investigation of duplicate handling via overall record averaging and averaging of cell line-specific means was performed. Within the 89 records of verapamil, 21 unique cell lines exist. However, the activities obtained from the two duplicate averaging strategies differed slightly (i.e., 5.99 vs 5.89). This is also observed for Ko143 (BCRP data set) and cyclosporine (MRP1 data set), with observed differences of less than 0.2 when comparing overall mean of reported activities and mean of cell line mean activities (e.g., Ko143: 6.88 vs 7.04, cyclosporine: 5.49 vs 5.38). This suggests that assay harmonization during curation does not have a significant impact on data consistency in the current heterogeneous transporter inhibition data sets. Therefore, duplicates were averaged uniformly across all records, with the final modeling sets consisting of 1,302, 1,010, and 368 unique compounds for P-gp, BCRP, and MRP1 (Figure S1).

External prediction data set cleaning followed the same curation procedure. The original DrugBank data set contained 12,309 drugs, which was reduced to 11,377 unique drugs after curation. After removing those present in the training data, the DrugBank prediction set consisted of approximately 11,300 new drug compounds. The unprocessed external Inxight Drugs set contained 13,455 records across 2,844 drugs, with 2,733 unique drugs remaining after curation. Similar to DrugBank, drugs present in the training set were removed from the Inxight Drugs set, resulting in an Inxight Drugs prediction set of 2,250 compounds.

### QSAR Model Development and Performance

For each transporter set, 17 models were built: 16 classic QSAR models with unique pairings between each ML algorithm (RF, SVM, XGB, *k*NN) and each chemical feature set (ECFP6, FCFP6, MACCS, RDKit), and one GCN model. The hyperparameter and configuration optimization search spaces for the classic ML algorithm hyperparameters and the GCN models are provided in Table S3. A t-distributed Stochastic Neighbor Embedding (t-SNE) visualization of the random-split training and test set chemical space shows that both the training and test set sufficiently represent the current data (Figure S3). Evaluation of activity-based and Kennard-Stone data splitting approaches showed no significant change in model performance compared to random splitting (Figure S4A), demonstrating the robustness of the current modeling framework across various splitting approaches. However, when employing a scaffold-based splitting approach, there was a noticeable drop in model performance for P-gp and BCRP, but not MRP1. This may be due to the polyspecificity of these transporters as well as the high diversity of the larger P-gp and BCRP modeling sets compared to MRP1. For example, following scaffold splitting, the nearest neighbor chemical similarity (ECFP6) for MRP1 is 15 to 20% higher than P-gp and BCRP. The P-gp and BCRP model test set performance, as a result, was negatively impacted by the diversity in the modeling sets when using the scaffold splitting approach. Alternative chemical descriptor families, such as Mordred descriptors, showed no significant difference in model performance to those used in the developed models (Figure S4B). Furthermore, the implementation of feature selection during model development (e.g., recursive feature selection and variance threshold feature selection) showed no significant impact on model performance (Figure S4C). The performance of different GCN models showed little variation across different layer configurations; thus, a representative 3-layer GCN model was selected by balancing computational time and model performance. A total of 51 individual QSAR models were built. Consensus models (generated by averaging individual model predictions) can reduce model-specific biases and improve model robustness in two ways: (1) by retaining predictions from diverse model settings, including different ML algorithms and chemical representations; (2) through the mitigation of positive and negative prediction errors from individual models when predictions are averaged. A comparison of different consensus averaging methods, including uniform consensus (each model contributes equally), weighted consensus (model contributions are weighted based on individual performance), and high-performing consensus (only high-performing models contribute), showed no significant changes in consensus model performance (Figure S4D). Thus, equal weights were assigned to each constituent model to construct the consensus models. Individual and consensus model 5FCV and test set performances are shown in Table 1.

**Table 1:** Individual and Consensus Model R^2^ Performances^a^

Algorithm	Descriptor	Coefficient of Determination (R^2^; Mean ± Standard Deviation)					
		P-gp		BCRP		MRP1	
		5FCV	Test	5FCV	Test	5FCV	Test
RF	ECFP6	0.61 ± 0.06	0.57	0.57 ± 0.07	0.54	0.67 ± 0.09	0.72
	FCFP6	0.58 ± 0.06	0.58	0.56 ± 0.05	0.50	0.66 ± 0.07	0.72
	MACCS	0.61 ± 0.04	0.62	0.53 ± 0.04	0.48	0.58 ± 0.06	0.76
	RDKit	0.58 ± 0.03	0.54	0.56 ± 0.04	0.54	0.65 ± 0.08	0.77
SVM	ECFP6	0.65 ± 0.04	0.62	0.57 ± 0.04	0.53	0.63 ± 0.08	0.59
	FCFP6	0.64 ± 0.02	0.59	0.59 ± 0.06	0.55	0.62 ± 0.09	0.70
	MACCS	0.61 ± 0.07	0.61	0.50 ± 0.07	0.55	0.61 ± 0.07	0.68
	RDKit	0.61 ± 0.04	0.64	0.52 ± 0.05	0.51	0.65 ± 0.08	0.76
XGB	ECFP6	0.64 ± 0.03	0.59	0.56 ± 0.07	0.45	0.61 ± 0.09	0.66
	FCFP6	0.62 ± 0.03	0.58	0.57 ± 0.07	0.48	0.63 ± 0.09	0.66
	MACCS	0.59 ± 0.04	0.57	0.47 ± 0.07	0.33	0.49 ± 0.12	0.72
	RDKit	0.61 ± 0.02	0.60	0.51 ± 0.08	0.50	0.62 ± 0.11	**0.78**
*k*NN	ECFP6	0.60 ± 0.03	0.52	0.48 ± 0.04	0.47	0.63 ± 0.07	0.69
	FCFP6	0.59 ± 0.04	0.52	0.46 ± 0.08	0.47	0.55 ± 0.15	0.56
	MACCS	0.57 ± 0.10	0.58	0.39 ± 0.11	0.49	0.54 ± 0.10	0.74
	RDKit	0.60 ± 0.02	0.63	0.44 ± 0.06	0.40	0.59 ± 0.08	0.72
GCN	Graphs	0.56 ± 0.04	0.53	0.49 ± 0.05	0.52	0.49 ± 0.09	0.69
Consensus	-------------	**0.70 ± 0.03**	**0.68**	**0.62 ± 0.05**	**0.58**	**0.68 ± 0.07**	**0.78**

a Best performing 5-fold cross validation (5FCV) and test set model R^2^ for each transporter are shown in bold.

The 5FCV R^2^ for individual models ranged from 0.39 to 0.67, with 5FCV mean absolute error and root mean squared error values ranging from 0.37 to 0.49 and 0.51 to 0.65, respectively. The external test set predictions showed similar model performance (Table 1, Table S4, and Table S5), indicating the reliability of the models for predicting new compounds. Except for the MRP1 test set predictions, consensus models outperform every individual ML and GCN model across the 5FCV and test set R^2^ performances. Moreover, the consensus models achieve an R^2^ improvement of 0.17–0.33 compared to Tanimoto similarity-based models, where the reported activity of the most similar training set compound was set as the predicted activity of a given test compound. This is consistent with previous studies in which consensus models achieved comparable or improved performance over individual models.^64,65,79–81^ Note, despite relatively modest individual performance, the graphic knowledge offered by the GCN models provides an alternative perspective to the classic descriptor-based ML models, positively impacting consensus model performance across all transporters, overcoming biases present in classic ML models. Upon comparison, these models also demonstrate superior performance over the Transporter Interaction Prediction (TIP) online web resource,^82^ a recently published online resource providing inhibitory potency predictions. Specifically, consensus models outperformed TIP model performance (5FCV R^2^) by an average of 8.1% (P-gp: 5.9%, BCRP: 11.1%, MRP1:7.2%). Thus, consensus models were selected for external predictions of new compounds.

The comparison of consensus predictions and observed IC_50_ values for both 5FCV and external test set compounds is shown in Figure 2. On average, 51% of 5FCV compounds were accurately predicted within 2-fold error, while 86% of the 5FCV predictions were acceptable, falling within 5-fold error. Similarly, 85% to 90% of test set compounds had acceptable predictions within 5-fold error, which further demonstrates the stability of the models, as similar results were achieved for both 5FCV and test set predictions.

**Figure 2:**
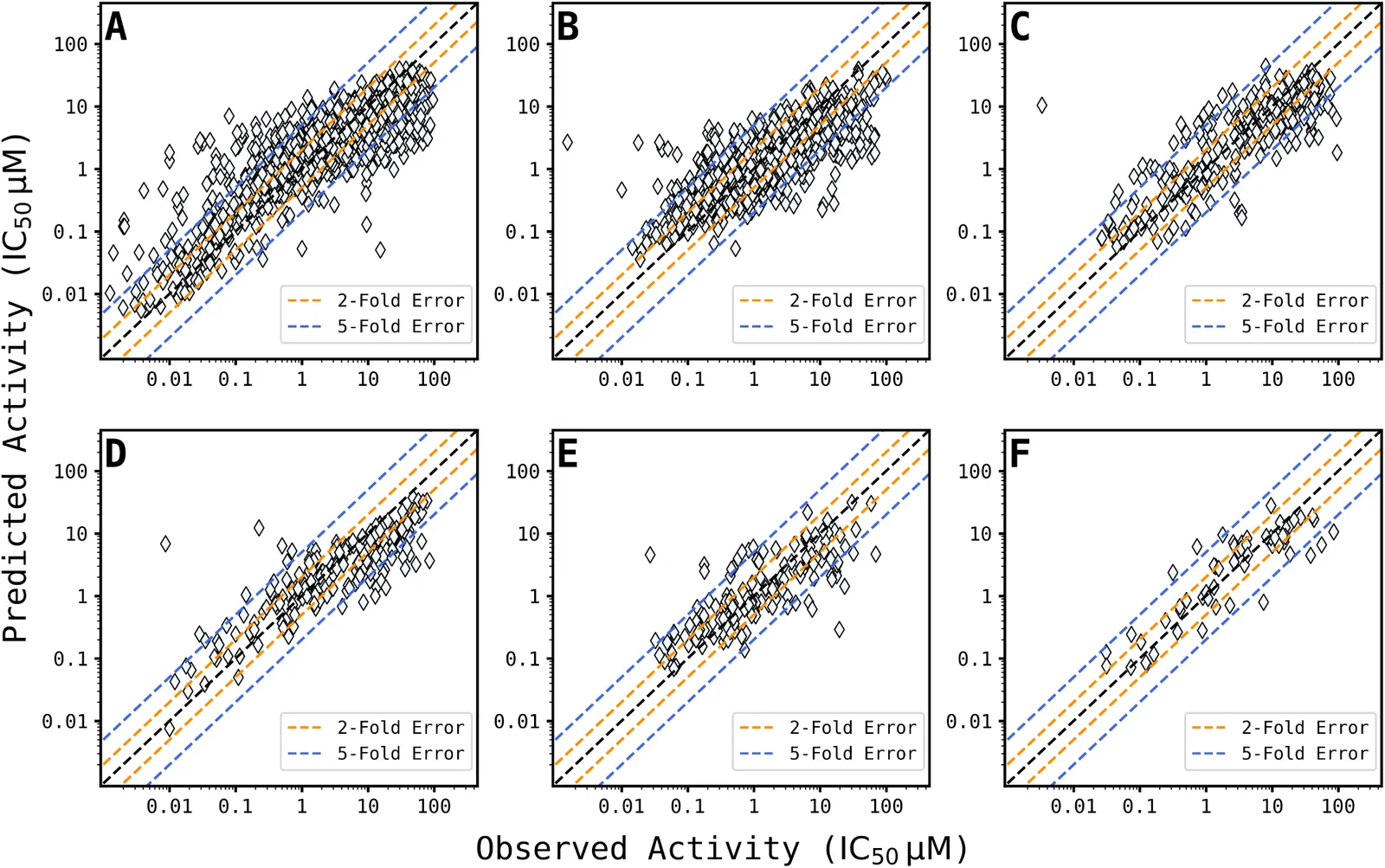
Observed versus predicted IC_50_ activities in the (A–C) 5FCV and (D–F) test set predictions for P-gp, BCRP, and MRP1, with 2-fold (orange dashed lines) and 5-fold (blue dashed lines) error visualization. Consensus model predictions shown.

Although promising correlations between predicted and observed IC_50_ values are shown in Figure 2, several compounds with underpredicted activities exist. Prediction deviations mainly arise from two sources: (1) test compounds lying outside of the model’s AD (i.e., structurally dissimilar from the training set); (2) systemic experimental discrepancies across different laboratories generating public IC_50_ data. In this study, we applied a chemical similarity-based AD to gain insights into these prediction error cases. The effect of various AD thresholds on model performance and chemical-space coverage is shown in Figure 3. Using Tanimoto similarity to calculate the similarity between test set compounds and their nearest neighbors in the training set, a test set compound is removed from the predictions if the similarity to its nearest training set neighbor is below a given threshold (Figure 3). The results show that when more chemicals are excluded from the test set by applying stricter AD thresholds, most models showed higher prediction accuracy (higher R^2^ values). This trend is consistent with what we obtained in our previous studies and can potentially be used to select new chemicals for experimental testing. Based on these results, an ECFP6 AD threshold of 0.4 was selected for subsequent analyses, balancing the improved predictive accuracy with the decreased coverage of the chemical space (i.e., number of test chemicals in the AD). This threshold retained approximately 90% of the test set chemicals and improved predictive accuracy (R^2^) by 7%. At more restrictive thresholds, improvements in model performance are accompanied by substantial decreases in chemical-space coverage. Alternative threshold selection may prove beneficial at different stages of drug development and compound investigations, such as prioritizing wider coverage during virtual screening or higher confidence during hit optimization.

**Figure 3:**
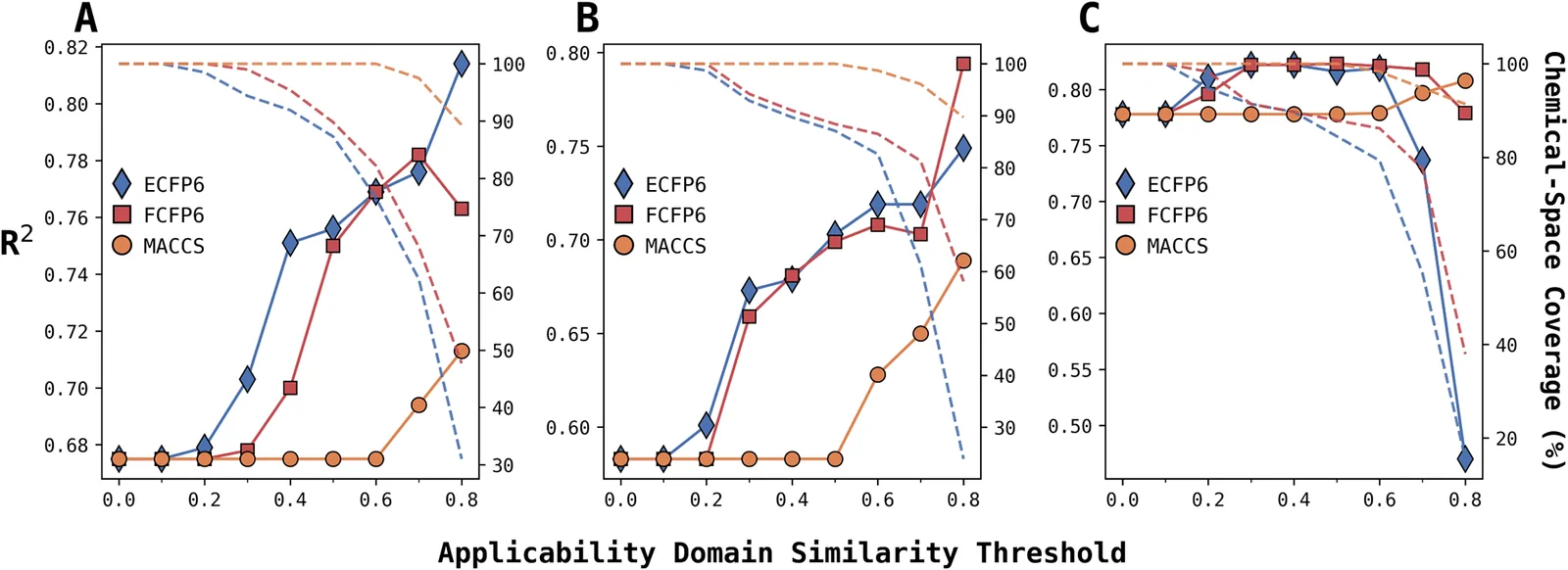
Consensus model performance and chemical-space coverage for (A) P-gp, (B) BCRP, and (C) MRP1 test predictions at different AD similarity thresholds. ECFP6 (blue, diamond), FCFP6 (red, square), and MACCS (orange, circle) Tanimoto similarity scores were calculated for each test set chemical and its nearest neighbor in the relevant training set. Test set chemicals with calculated similarities below a given threshold are excluded, depicted by the drop in coverage (dashed lines).

It is worth noting that the only exception (i.e., prediction accuracy decreased with more restrictive AD thresholds) is observed for MRP1 at high AD thresholds (Figure 3C). The MRP1 modeling set contains only 368 unique compounds (310 training and 58 test), leading to insufficient coverage of the overall chemical space. When high Tanimoto thresholds are applied, only a small fraction of MRP1 test compounds remains for evaluation, potentially introducing statistical bias. Therefore, the application of an AD is also determined by the chemical space coverage of the training set (i.e., size and diversity of training set compounds). To enhance MRP1 model robustness, future efforts should prioritize expanding the size and diversity of the MRP1 bioactivity data set.

The observed relationship between structural similarity and prediction accuracy suggests that some errors may be due to outliers in the training data. Structurally dissimilar underpredicted compounds can be handled by applying an AD; however, structurally similar activity cliffs also exist. We speculated that these errors could be due to differences in experimental protocol (e.g., cell line or substrate used), yielding more sensitive results for the observed outliers. Thus, we selected eight training compounds (four P-gp and four BCRP inhibitors), which induced activity cliffs (i.e., causing large prediction errors of structurally similar compounds), to be retested to confirm whether their prediction errors were due to model performance or potential experimental errors in the original reports. We found that, for the majority of cases, the previously reported activities (i.e., training set IC_50_ values) were significantly more potent than in our experiments (395-fold difference in potency on average). Detailed information for these eight compounds, including the original training data and new experimental results using transporter-overexpressing cells, is provided in Table S6. Additionally, after incorporating the updated experimental activities, model predictions greatly improved. For example, the predictions for febuxostat (CHEMBL1164729) and ONT-093 (CHEMBL313113) improved to fold errors of 3.6 and 1.5 with the updated training data (a 45x and 80x reduction in fold error, respectively). Furthermore, after updating febuxostat’s activity, the BCRP test set R^2^ increased from 0.58 to 0.62. This suggests that potential errors (i.e., caused by protocol variation or experimental errors) in the training sets negatively impact model performance.

Systemic deviations in reported IC_50_ values originating from inconsistent *in vitro* experimental protocols are a common artifact found in large public bioactivity databases, which can result in large prediction errors. When possible, standardized retesting and data set refinement can serve as critical preprocessing steps to improve QSAR model accuracy. Future cheminformatics studies relying on public bioactivity resources may benefit from similar retesting procedures. Alternatively, to leverage all publicly available bioactivity data, harmonization of conflicting experimental protocols requires further attention. ML approaches which can reflect data inconsistencies require clear definition and standardization of experimental conditions, which are currently lacking across many public records and databases. Ongoing efforts by our lab to construct a modeling framework incorporating experimental variables to reflect different experimental systems require the reporting of sufficient experimental information.

### Identification of Key Structural Features Influencing Model Predictions

To identify key substructures accounting for ABC transporter inhibitions, Shapley Additive Explanations^83^ (SHAP) analysis was performed as previously reported^57^ to select the top descriptors for IC_50_ predictions. Briefly, using MACCS descriptors and the RF algorithm, the top MACCS descriptors were identified for P-gp, BCRP, and MRP1 inhibition modeling (Figure S5). In addition to SHAP, local interpretable model-agnostic explanations (LIME) analysis of the molecular graphs was performed using a predefined set of 83 functional groups to identify molecular substructures and frameworks with the most impact on GCN. The LIME algorithm allows model predictions to be explained based on interpretable representations through local approximation.^84^ Key global trends affecting model predictions were revealed through the aggregation of functional groups that contributed the most to the model predictions across the transporter data sets (Figure S6).

The SHAP-identified top descriptors for P-gp suggest that P-gp inhibition is significantly affected by oxygen-containing groups. LIME-identified functional groups are consistent with these findings, where various hydrogen bond acceptors and donors and hydrophobic substructures are among the most impactful. These results resonate with previous evaluations of P-gp binding and inhibition,^85,86^ and are found in well-known inhibitors such as tariquidar and verapamil. Alternatively, BCRP SHAP analysis determined that nitrogen-containing groups, such as heterocycles and N–H groups, had a large impact on model predictions. Functional groups identified through LIME analysis also showed that top contributors include aniline, tetrazole, nitro, and pyridine, echoing previous studies exploring BCRP inhibition by 4-anilino-quinazolines and -quinolines^87^ or benzamide and phenyltetrazole derivatives,^88^ as well as potent inhibitors such as Ko143 and elacridar. Finally, SHAP analysis of MRP1 inhibition found 5-membered heterocycles and halogens to impact model predictions significantly. Among the top LIME functional groups, thiophene and halogen are consistent with the SHAP findings. Avapritinib, a potent MRP1 inhibitor in the current data set, and indomethacin, a selective MRP1 inhibitor,^89^ both contain these 5-membered heterocyclic and halogenated structures. Past studies also investigated MRP1 inhibitors containing 5-membered heterocyclic frameworks, such as thiophenes and isoxazoles.^90,91^

### Virtual Screening of DrugBank Compounds for Experimental Validation

The developed consensus models were used to screen approximately 11,300 curated DrugBank chemicals. Due to the limitations of the MRP1 data set in the present work, the MRP1 model is not suitable for high-confidence external predictions and serves only as a proof-of-concept for future studies once additional data becomes available. As such, virtual screening efforts were only applied to the P-gp and BCRP models. Model predictions were used to rank chemicals for their probability to be P-gp or BCRP inhibitors (i.e., higher potency, as indicated by lower IC_50_ values, corresponds to higher likelihood of being an inhibitor). Drug categories provided by DrugBank, such as “P-glycoprotein Inhibitors” and “BCRP/ABCG2 Inhibitors”, were used to label external DrugBank chemicals as inhibitors or non-inhibitors. Figure 4A shows that the P-gp and BCRP model predictions can identify inhibitors with greater confidence than random or permuted selections. The P-gp and BCRP predictions can achieve respective enrichments of 16-fold and 35-fold among the top ranked chemicals (Figure 4B). Overall, our models successfully identified inhibitors (as reported by DrugBank) of P-gp (e.g., elacridar, dofequidar, and hycanthone) and BCRP (e.g., ripretinib, capmatinib, and tepotinib) and can lead to the future identification of potential inhibitors.

**Figure 4:**
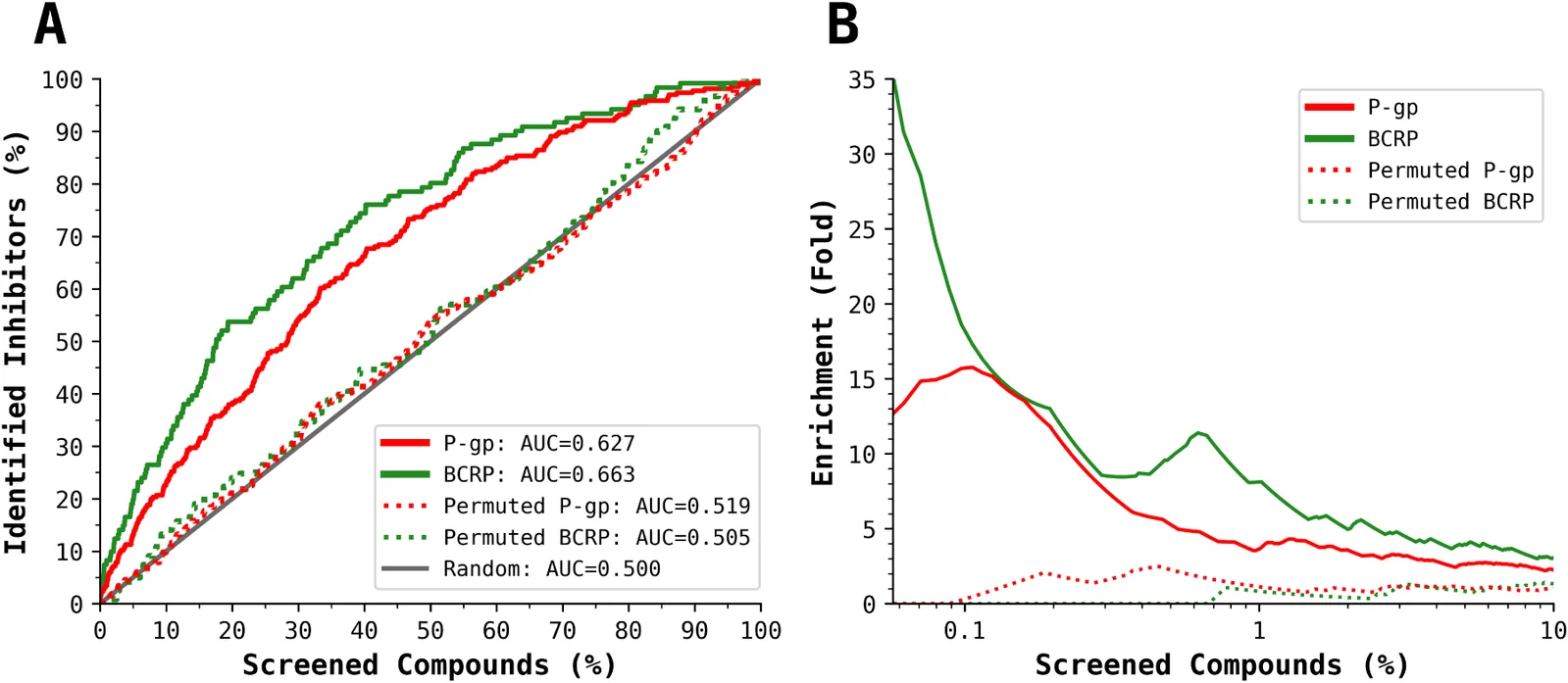
Identification of P-gp and BCRP inhibitors in the DrugBank external set by consensus model predictions: (A) cumulative identification of inhibitors across 11,300 DrugBank compounds; and (B) enrichment within the top ranked candidates. Random and permuted results included for comparison.

We selected 22 DrugBank compounds, which did not exist in our training set, for experimental validation. The selected chemicals represent a range of predicted activities and diverse structures. Among these chemicals, four have DrugBank transporter inhibition results (i.e., labeled as P-gp or BCRP inhibitors) and the remaining were unknown compounds. Some selected compounds were mapped and shown in P-gp and BCRP training set chemical spaces in Figure 5. The t-SNE plots were generated with the scikit-learn library by using MACCS fingerprints and Euclidean distances. Here, we can visualize the training set compounds that each selected DrugBank compound is most closely related to within the defined chemical space. Lucanthone and naftopidil appear near clusters of high potency, while cases such as ilginatinib and elenestinib appear within larger training set clusters of diverse activities.

**Figure 5:**
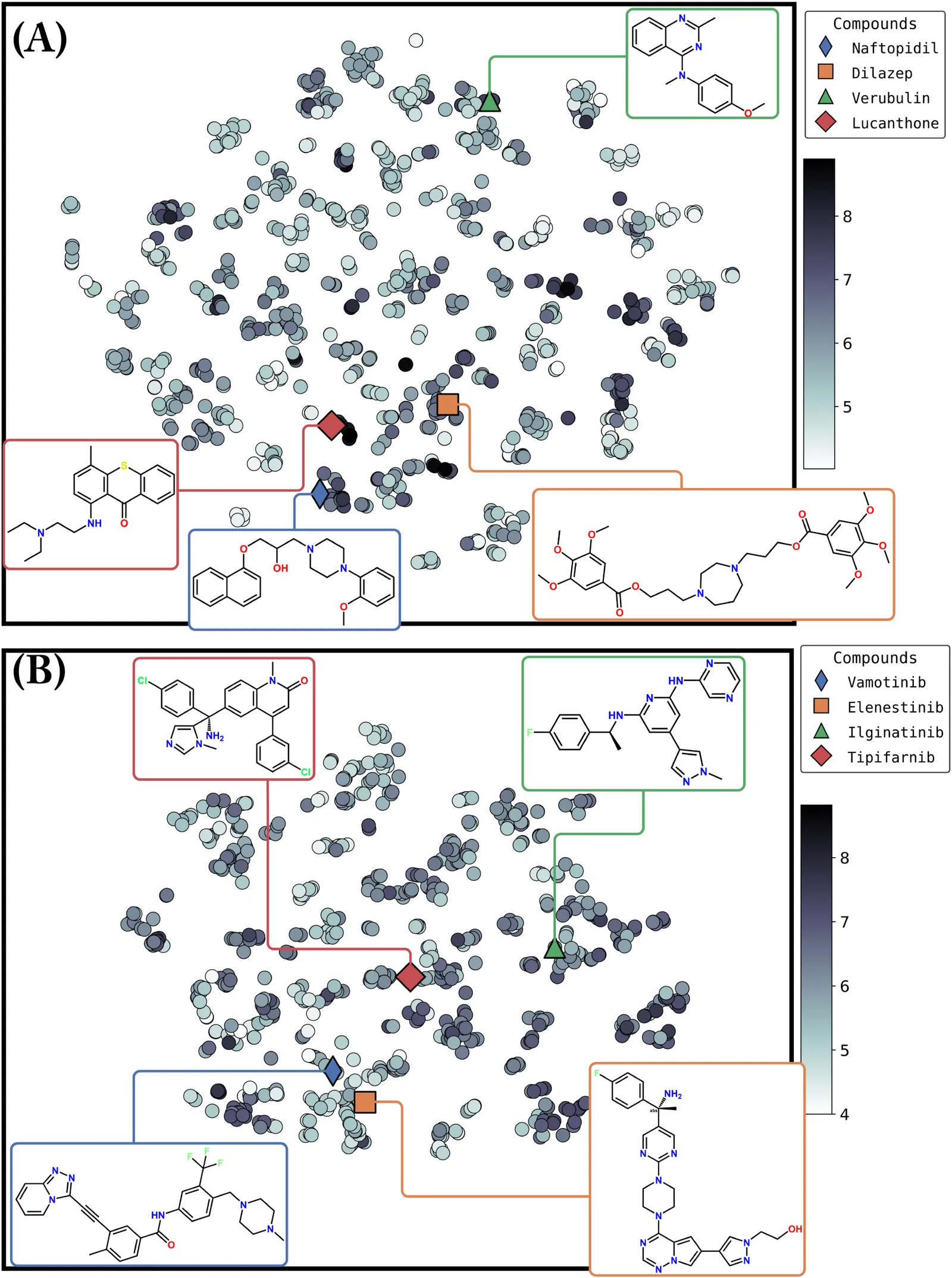
t-SNE visualization of selected DrugBank chemicals within (A) P-gp and (B) BCRP training set chemical spaces. Training compound points are colored by their normalized pIC_50_ (−log­(IC_50_)) values.

Detailed information for the 22 selected chemicals is shown in Table 2. Among these chemicals, 16 were predicted to be potential high-potency inhibitors (0.08 μM ≤ IC_50_ ≤ 1.67 μM), with the remaining six predicted as low-potency/non-inhibitors (IC_50_ > 10 μM). The experimental testing showed that, of the 12 drugs with measured IC_50_ values, those within the AD had an average fold-error of 2.5, while those out of domain had an average fold-error of 12.7. Furthermore, model predictions yielded an R^2^ score of 0.72 for external chemicals within the AD. More restrictive AD thresholds saw further improvements in predictive accuracy, with R^2^ values surpassing 0.8 at thresholds above 0.4; however, few compounds remain within the chemical space of the AD.

**Table 2:** Detailed Information for 22 Experimentally Tested DrugBank Chemicals^a^

	Drug Name	Predicted IC_50_	Experimental IC_50_	Max. Training Sim.	In AD
P-gp	Lucanthone	0.41	–	0.22	No
	Ranolazine	0.71	–	0.40	Yes
	Naftopidil	0.78	–	0.43	Yes
	Verubulin	0.75	–	0.39	No
	Dofequidar	0.75	0.56	0.51	Yes
	Dilazep	1.06	3.65	0.29	No
	Ethylparaben	11.92	–	0.23	No
	Hydroquinone	28.53	–	0.33	No
	Piceatannol	43.12	–	0.53	Yes
BCRP	Elenestinib	0.08	0.355	0.77	Yes
	Pranlukast	0.66	–	0.27	No
	Vamotinib	0.51	0.26	0.63	Yes
	Tipifarnib	1.03	6.70	0.19	No
	Belumosudil	1.06	0.26	0.48	Yes
	RAD-140	1.04	15.80	0.19	No
	Verucerfont	1.01	15.50	0.21	No
	Ilginatinib	1.25	0.715	0.30	No
	Mobocertinib	1.53	7.60	0.26	No
	Preladenant	1.67	61.88	0.21	No
	Cabotegravir	14.56	–	0.77	Yes
	Rifapentine	18.14	29.05	0.85	Yes
	Aranidipine	18.85	–	0.80	Yes

a AD threshold of 0.4 (ECFP6).

As shown in Table 2, the IC_50_ values for several predicted high-potency inhibitors could not be experimentally detected in P-gp or BCRP-overexpressing cells. Based on chemical structures and available literature, it is likely that various false negatives exist using this fluorescent substrate accumulation assay. For example, the IC_50_ of ranolazine, a known P-gp inhibitor with reported clinical DDIs, could not be determined. A key limitation of this assay is that cis-inhibition requiring active uptake of the putative inhibitor is unlikely due to the low basal expression of influx carriers. Rather, this approach will be able to detect chemicals with trans-inhibition of P-gp or BCRP or inhibitors that use passive permeability to enter cells. Recognizing this limitation, we explored the utility of inverted membrane vesicles generated from cells expressing BCRP as a proof-of-concept. Using this alternative inverted vesicle approach, pranlukast, preladenant, and aranidipine were confirmed as inhibitors of BCRP at 50 and 100 μM despite little to no inhibition in cell-based assays. Overall, experimental testing on drug molecules selected by virtual screening confirmed the capability of the P-gp and BCRP models to identify ABC transporter inhibitors. For example, vamotinib, elenestinib, and ilginatinib were identified as potent BCRP inhibitors, with respective predicted and experimental IC_50_ values of 0.51/0.26 μM, 0.08/0.355 μM, and 1.25/0.715 μM in cell-based assays. With only 16 drugs selected for testing from the external DrugBank set, further virtual screening using the developed models would likely identify additional novel inhibitors of these ABC transporters.

The DrugBank virtual screening results underscore the challenges of data harmonization and the differences in observed activities across differing experimental conditions. In the overexpressing-cell system, the inhibitory potency IC_50_ can be affected by several factors, including cellular uptake, membrane permeability, passive diffusion, intracellular distribution, and metabolism. In contrast, the inverted membrane vesicle assay minimizes these intracellular variables and provides a more direct assessment of compound activity under the defined assay conditions. When used to complement one another, these two experimental approaches can reduce the possibility of false-negative results, as demonstrated here. However, observations of cross-assay variability draw further attention to the need for public data to clearly report key experimental conditions so that more informed predictive models can be developed.

### DDI Risk Evaluation Using Established Models

The established P-gp and BCRP models were used to evaluate 2,250 Inxight Drugs compounds. Using model-predicted IC_50_ and reported *C*_max_ values, the DDI Ratio was calculated for each target compound. eq 1 presents the recommended ratio calculation to evaluate DDI risk based on FDA industry guidelines.^10^ Accordingly, eq 1 was incorporated into our DDI risk assessment procedure. Beyond the predicted ratios, drug candidates must also be evaluated for their likelihood of being an inhibitor of P-gp or BCRP. Thus, we employed our in-house classification models to generate inhibitor probability predictions.^57^

Compared to this study, the actual FDA guidance uses unbound *C*_max_ (*C*_max,u_) and unbound IC_50_ (IC_50,u_) in the DDI Ratio. When this ratio exceeds 0.02, DDI risk should be experimentally investigated further. However, many drugs lack sufficient pharmacokinetic data to incorporate fraction unbound in this work. Once relevant experimental data is available, the same strategy can be deployed after retraining the models with the expanded data. To address this discrepancy, the FDA-defined cutoff value of 0.02 was used as a lower threshold for Ratio Scores of two or higher (Table S2). This threshold requires the predicted ratios to exceed the FDA-defined cutoff to achieve a DDI risk label of moderate or high when accompanied by a high-confidence inhibitor probability prediction (e.g., probability ≥ 60%). Furthermore, by including predicted inhibitor probabilities, drugs which are confidently predicted as non-inhibitors (e.g., probability ≤ 20%) are labeled Low Risk, as the drug would not cause a DDI as a non-inhibitor. In this way, both the potency and likelihood of inhibition are incorporated into the DDI risk assessment protocol. The sum of the Ratio Score and Probability Score yielded the Total Score used to designate drugs as low, moderate, or high-risk inhibitors causing P-gp- or BCRP-mediated DDIs. Risk label distributions for 2,250 Inxight Drugs compounds are shown in Figure 6A, with the distribution of predicted ratios and probabilities for P-gp and BCRP shown in Figure 6B and C. To address the impact which potential prediction errors may have on DDI risk assessment, 90% prediction intervals were determined from 5FCV model predictions and subsequently validated on test set predictions. Sensitivity analysis of DDI risk labels to these prediction intervals found that approximately 73% of Inxight Drugs chemicals had no change in predicted risk label. For the remaining 27%, 25% crossed either the low-moderate threshold or moderate-high threshold, and less than 2% transitioned between low and high risk. These findings suggest reasonable stability of the DDI risk labels even when prediction intervals are considered, but further analysis is required for compounds which are close to the defined thresholds and susceptible to the prediction intervals.

**Figure 6:**
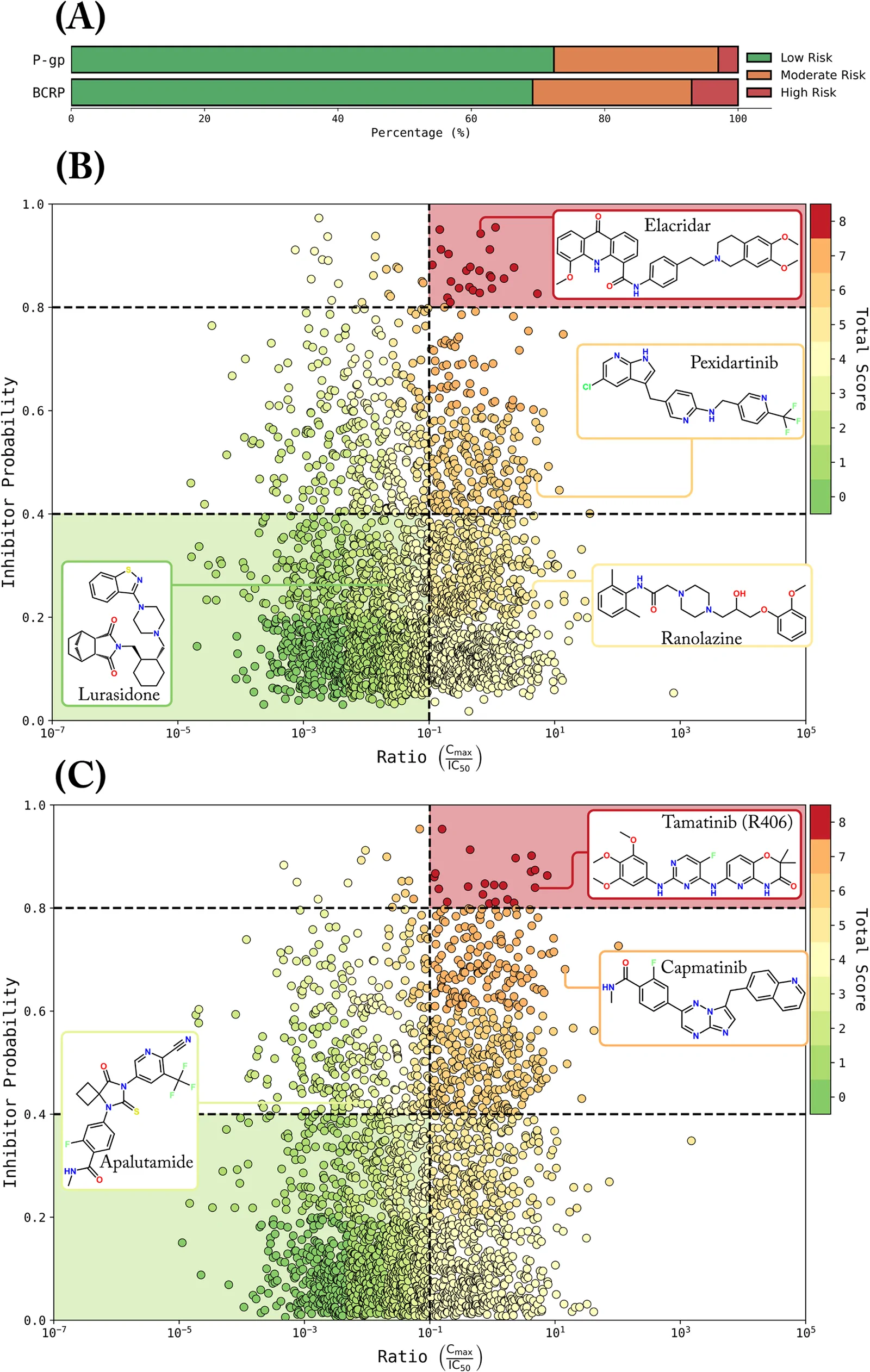
Evaluation of 2,250 Inxight Drugs compounds for their risk of DDI: (A) stacked bar chart representing risk label percentages in P-gp and BCRP predictions. Ratio versus probability scatterplots for (B) P-gp and (C) BCRP, with associated total scores designated by color. Structures and locations of example drug compounds are shown.

To evaluate our protocol’s ability to identify perpetrators of P-gp- and BCRP-mediated DDIs, we obtained reported DDIs from the FRDB DDI data set provided by Inxight Drugs.^60^ Our proposed DDI risk assessment strategy demonstrated a strong ability to identify DDI perpetrators. For example, elacridar and tamatinib were labeled as high-risk P-gp and BCRP inhibitors (Figure 6B and C; both with Total Scores of 8). Elacridar is a well-known P-gp inhibitor, with previous reports of intestinal, brain, and placental DDIs.^92–98^ These studies report significantly altered accumulation of P-gp substrates such as methadone, acyclovir, and digoxin. For example, Sugimoto et al. reported that elacridar increased the digoxin brain-to-plasma concentration ratio 14-fold compared to the control in rats.^96^ Tamatinib, the active metabolite of fostamatinib, was predicted as a high-risk DDI inhibitor of BCRP by our evaluation system. Tamatinib is known to alter BCRP substrate area under the concentration–time curve (AUC) and *C*_max_ and enter systemic circulation in its prodrug form.^99^ Several moderate-risk inhibitors were also identified by our protocol. For example, ranolazine (Total Score of 5: ***R*** = 4, ***P*** = 1), implicated in a clinical DDI affecting digoxin pharmacokinetics,^100^ and pexidartinib (Total Score 6: ***R*** = 4, ***P*** = 2), with a reported DDI with digoxin that is consistent with intestinal P-gp inhibition (according to FRDB DDI data), were labeled as moderate-risk inhibitors.

A number of previously reported *in vitro* and/or *in vivo* inhibitors with no reported clinical DDIs in the FRDB DDI data set involving P-gp (e.g., tetrandrine,^101^ cepharanthine)^102^ or BCRP (e.g., dacomitinib,^103^ xanthohumol)^104^ were predicted to cause DDIs. Additionally, there are several high-risk cases with no previous reports of inhibition, such as deserpidine and ridaforolimus (high-risk P-gp cases), and radotinib and tesevatinib (high-risk BCRP cases). Deserpidine, an antihypertensive with antipsychotic properties, and ridaforolimus, a mammalian target of Rapamycin (mTOR) inhibitor, are structurally similar to reserpine and rapamycin, which are established P-gp inhibitors, substrates, and inducers, according to DrugBank.^59^ Radotinib is a second-generation tyrosine kinase inhibitor with a high structural similarity with nilotinib,^105^ a tyrosine kinase inhibitor that potently inhibits BCRP at clinically relevant concentrations. Of particular interest to BCRP-mediated DDI risk evaluation are epidermal growth factor receptor (EGFR) inhibitors. Several EGFR inhibitors have previously been reported to inhibit BCRP, including erlotinib^106^ and gefitinib^107^ (neither present in the Inxight Drugs external set), as well as osimertinib^108^ and allitinib^109^ (high-risk BCRP cases). Notably, tesevatinib,^110^ a potent EGFR inhibitor with no reported inhibition of BCRP, was also predicted to cause BCRP-mediated DDIs. Accordingly, these potential DDI-perpetrating chemicals should be further studied when they are administered with relevant P-gp or BCRP substrates.

Further investigation of known DDI perpetrators shows noticeable differences among the reported alterations of substrate pharmacokinetics across the defined risk labels, showing that the models can potentially distinguish between DDIs of different magnitudes (Table 3), in addition to DDI perpetrators and non-perpetrators. For example, elacridar, telaprevir, and lurasidone, as representatives of the high-risk, moderate-risk, and low-risk drugs by our evaluation system, result in respective P-gp substrate *C*_max_ increases of 180%, 50%, and 9.4%. This is evidenced further by tamatinib, capmatinib and apalutamide for BCRP, or when looking only at DDI perpetrators within the AD, such as elacridar and cobicistat for P-gp. Tamatinib and capmatinib are both reported to considerably increase the *C*_max_ and AUC of rosuvastatin, a BCRP substrate, while the reported DDI for apalutamide describes a decrease in rosuvastatin AUC (Table 3). The interaction between cobicistat and digoxin causes only an 8% increase in digoxin AUC, a significantly weaker effect than that of elacridar (a 180% increase in AUC). Altogether, our DDI risk assessment protocol demonstrates the potential to identify perpetrators of transporter-mediated DDIs of varying magnitudes and differentiate between inhibitors capable of causing harmful DDIs and weak inhibitors unlikely to cause clinically relevant DDIs. Several limitations exist in the DDI risk analysis. Beyond fraction unbound, various pharmacokinetic determinants are not incorporated into the current strategy. This remains a limiting factor for early drug development efforts, since many chemicals lack this information. Considering the impact these determinants may have on the true conditions in which a DDI may occur, the current protocol may misrepresent DDI potential (e.g., overpredict the risk of highly protein-bound drugs) and should therefore not be used for direct assessment of real-world DDI outcomes. Rather, this approach serves as a strategy to quickly assess the risk of novel compounds perpetrating DDIs and can inform further interrogation of potential high-risk compounds. Based on the current work, future efforts with more comprehensive training data will likely expand the scope of the current risk assessment protocol (i.e., accurately differentiate the DDI magnitudes caused by different transporter inhibitors).

**Table 3:** Detailed Information for Selected Cases of Reported Drug–Drug Interactions

Drug	Transporter	Risk Label	Total Score	In AD^a^	Effect on PK of Victim Drug^b^
Elacridar	P-gp	High	8	Yes	180% increase
Telaprevir	P-gp	Moderate	5	No	50–85% increase
Cobicistat	P-gp	Low	3	Yes	8–41% increase
Lurasidone	P-gp	Low	3	No	9–13% increase
Tamatinib	BCRP	High	8	Yes	88–96% increase
Capmatinib	BCRP	High	7	No	108–204% increase
Apalutamide	BCRP	Low	3	No	41% decrease

a AD threshold of 0.4 (ECFP6).

b Reported change in substrate exposure (AUC or *C*_max_) according to the FRDB DDI data set.

In this work, we developed classic and graph-based QSAR models for three ABC transporter chemical inhibitions. The resultant models generated satisfactory predictions, with greatly improved performance when applying ADs. These models successfully identified potential inhibitors of P-gp and BCRP present in the DrugBank data set. Using the developed QSAR models, we constructed a novel DDI risk assessment protocol, which can directly evaluate new drug candidates for their DDI potential using their chemical structures. To our best knowledge, this effective assessment strategy can be applied to additional activities/properties of clinical importance and provide regulatory agencies a new scoring system to evaluate new drugs without experimental testing.

## Supplementary Material





## Data Availability

The data preprocessing and curation procedure (IPYNB), curated modeling data sets (SDFs), and source code for this project are publicly available at https://github.com/zhu-research-group/reg_qsar_gcn. The ABC transporter inhibition data used in this study, including chemical SMILES and their bioactivities, are also available in the Supporting Information (XLSX).

## Notes

Curated ABC transporter inhibition data for approximately 2,200 compounds across three modeling sets (XLSX)
Activity distribution of collected ABC transporter data sets; protein expression of BCRP and P-gp in overexpressing cells; individual scoring criteria for the ratio and probability scores used for DDI risk assessment; t-SNE visualization of training (circles) and test set (triangles) compounds in the ABC transporter modeling sets; search spaces for classic ML hyperparameter and GCN configuration optimization; comparison of model performances (R^2^) using various (A) data splitting approaches, (B) chemical descriptor sets, (C) feature selection methods, and (D) consensus modeling methods; individual and consensus model mean absolute error performances; individual and consensus model root mean squared error performances; comparison of activities of eight retested training set compounds using higher concentrations of test chemical up to 100 μM in transporter-overexpressing cells; top MACCS descriptors in the (A) P-gp, (B) BCRP, and (C) MRP1 random forest models; top global contributing functional groups identified through local interpretable model-agnostic explanations (LIME) analysis for (A) P-gp, (B) BCRP, and (C) MRP1 (PDF)
