# Supplementary material for: Evaluating Drug–Drug Interactions by Constructing Advanced Machine Learning Models of ABC Transporter Inhibitors
Source: J Chem Inf Model. 2026 Oct 26;66(21):14231–46. doi: 10.1021/acs.jcim.6c02847 (PMC13753517; doi:10.1021/acs.jcim.6c02847)
Supplement: Supplementary file 1 [file ci6c02847_si_002.pdf]

Supporting Information

Evaluating Drug-Drug Interactions by Constructing Advanced Machine Learning Models of ABC Transporter Inhibitors

Sean R. Carey[1,2], Xia Wen[3], Nada J. Daood[1,2], Elena Chung[1,2], Jacqueline B. Tiley[4], Lauren M. Aleksunes[3], Hao Zhu[1,2]*

[1]Center for Biomedical Informatics and Genomics, Tulane University School of Medicine, New Orleans, LA 70112, USA.

[2]Department of Chemistry and Biochemistry, Rowan University, Glassboro, NJ 08028, USA.

[3]Department of Pharmacology and Toxicology, Rutgers University, Piscataway, NJ 08854, USA.

[4]Division of Pharmacotherapy and Experimental Therapeutics, UNC Eshelman School of Pharmacy, University of North Carolina at Chapel Hill, Chapel Hill, NC 27599, USA.

Corresponding Author

Hao Zhu – *Center for Biomedical Informatics and Genomics, Tulane University School of Medicine, 1430 Tulane Avenue, New Orleans, Louisiana, USA 70112;* Telephone: (504) 988-3443; orcid.org/0000-0002-3559-6129, Email: hzhu10@tulane.edu

<u>**Table of Contents**</u>

Figure S1. Activity distribution of collected ABC transporter datasets.

Figure S2. Protein Expression of BCRP and P-gp in Overexpressing Cells.

Table S2. Individual scoring criteria for the ratio and probability scores used for DDI risk assessment.

Figure S3. t-SNE visualization of training (circles) and test set (triangles) compounds in the ABC transporter modeling sets.

Table S3. Search spaces for classic ML hyperparameter and GCN configuration optimization.

Figure S4. Comparison of model performances (R^2) using various **(A)** data splitting approaches, **(B)** chemical descriptor sets, **(C)** feature selection methods, and **(D)** consensus modeling methods.

Table S4. Individual and consensus model mean absolute error performances.

Table S5. Individual and consensus model root mean squared error performances.

Table S6. Comparison of activities of eight retested training set compounds using higher concentrations of test chemical up to 100 µM in transporter-overexpressing cells.

Figure S5. Top MACCS descriptors in the **(A)** P-gp, **(B)** BCRP, and **(C)** MRP1 random forest models.

Figure S6. Top global contributing functional groups identified through local interpretable model-agnostic explanations (LIME) analysis for **(A)** P-gp, **(B)** BCRP, and **(C)** MRP1.

Additional File: Excel spreadsheet containing Supplementary Table 1.

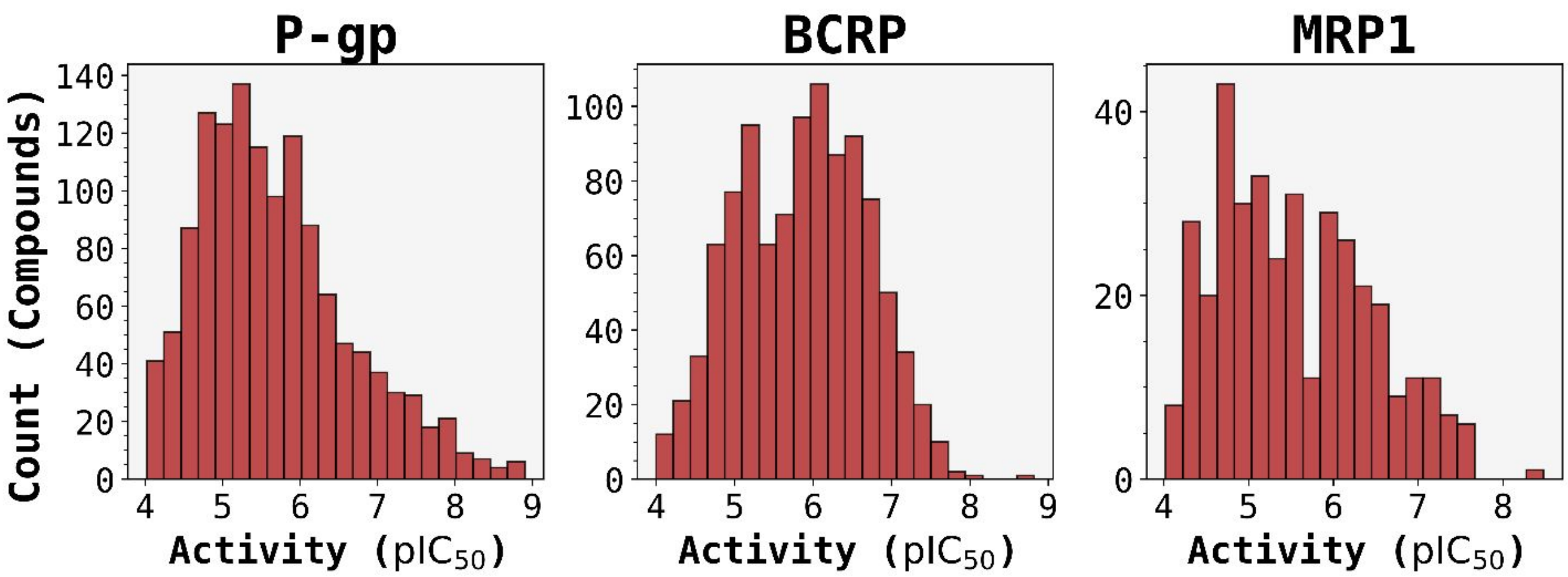

Figure S1. Activity distribution of collected ABC transporter datasets. pIC_{50} – negative log of half-maximal inhibitory concentration.

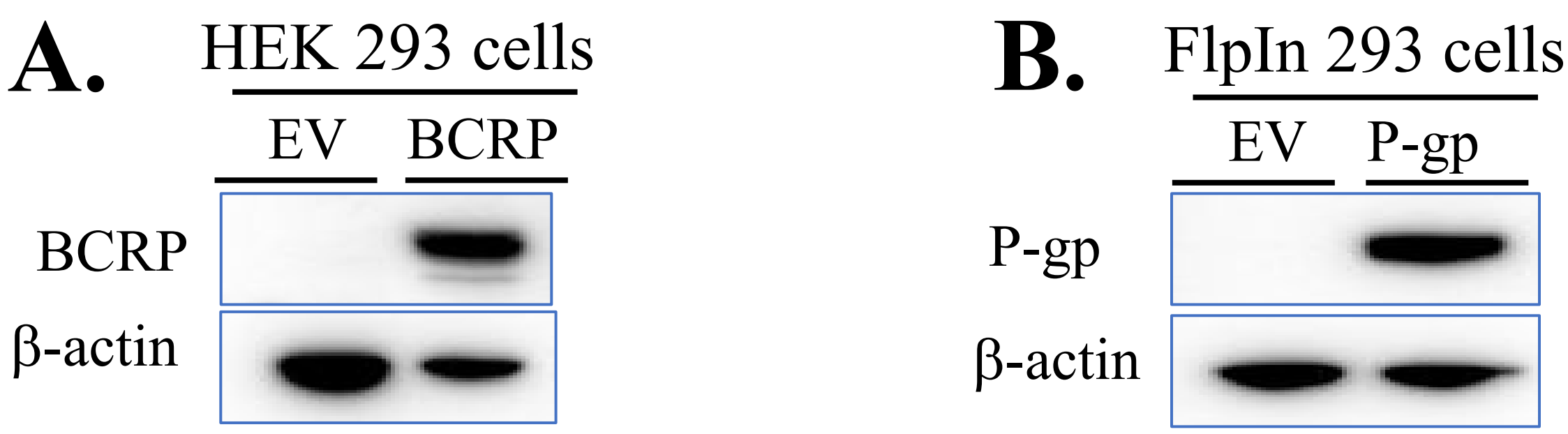

Figure S2. Protein Expression of BCRP and P-gp in Overexpressing Cells. The EV, BCRP or P-gp WT cells were lysed in lysis buffer containing 20 mM Tris-HCl, 150 mM NaCl, 5 mM EDTA, 1% Triton 100 and 1% protease inhibitor cocktail. The protein concentration of cell lysates was determined by the Bicinchoninic Acid (BCA) protein assay kit (ThermoFisher Scientific). cell lysates (15 µg of protein/sample) were separated by SDS-PAGE electrophoresis and transferred to a nitrocellulose membrane at 4°C overnight. After blocking with 5% nonfat dry milk in PBS with 0.5% of Tween 20 (PBS/T), membranes were incubated with primary antibody against **(A.)** BCRP (BXP-53, Abcam24115) or **(B.)** P-gp (C219) (Enzo Life Sciences, Farmingdale, NY) followed by incubation with anti-mouse secondary antibody (Sigma) for 1 to 2 h. The SuperSignal West Dura chemiluminescent substrate (ThermoFisher Scientific) was applied to membranes prior to detection of luminescence using a FluorChem E system (Protein simple, San Jose, CA). β-actin (Abcam, Cambridge, MA) was used as a loading control.

Table S2. Individual scoring criteria for the ratio and probability scores used for DDI risk assessment.

Individual Score	Individual Score Criteria	
	**Ratio Score**	**Probability Score**
**4**	$\frac{C_{max}}{IC_{50}} \geq 0.1$	Probability ≥ 0.8
**3**	$0.1 > \frac{C_{max}}{IC_{50}} \geq 0.05$	0.8 > Probability ≥ 0.6
**2**	$0.05 > \frac{C_{max}}{IC_{50}} \geq 0.02$	0.6 > Probability ≥ 0.4
**1**	$0.02 > \frac{C_{max}}{IC_{50}} \geq 0.005$	0.4 > Probability ≥ 0.2
**0**	$\frac{C_{max}}{IC_{50}} < 0.005$	Probability < 0.2

*IC_{50} –half-maximal inhibitory concentration; C_{max} – maximum plasma concentration.

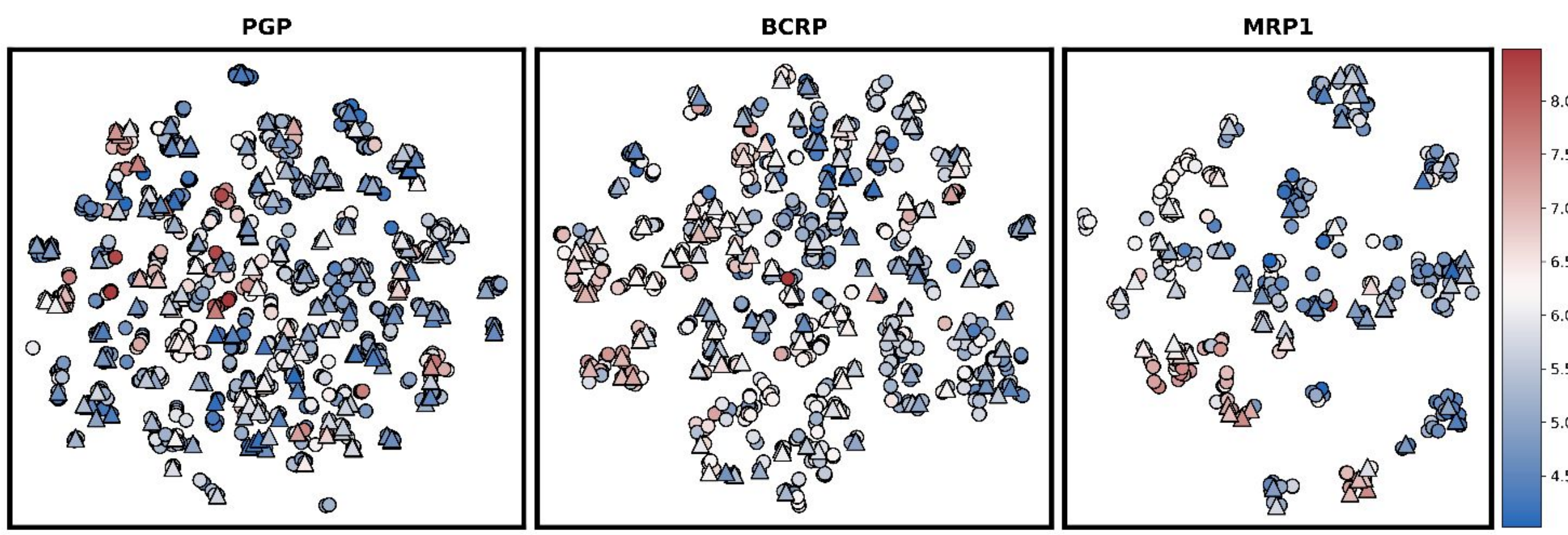

Figure S3. t-SNE visualization of training (circles) and test set (triangles) compounds in the ABC transporter modeling sets. Points are colored by their normalized pIC50 (-log(IC50)) values.

Table S3. Search spaces for classic ML hyperparameter and GCN configuration optimization.

Model	Parameter	Search Space
RF	n_estimators: number of estimators	5, 10, 25, 50, 100
SVM	gamma: kernel coefficient	1e-2, 1e-3
	C: regularization parameter	1, 10
*k*NN	n_neighbors: number of neighbors to use	1, 3, 5, 7, 10
	weights: weight function used in prediction	uniform, distance
GCN	gcn_layer_dimensions: size of graph convolutional layers	64, 96, 128, 256, 512
	n_layers: number of graph convolutional layers	1, 2, 3, 4, 5
	dropout: dropout probability for each layer	0.0, 0.1, 0.2, 0.3, 0.4, 0.5
	batch_size: number of samples processed per batch	16, 32, 64, 128
	n_epochs: number of iterations through full dataset	10, 25, 50, 100, 500
	learning_rate: magnitude of adjustments during optimization	0.001, 0.0001, dynamic

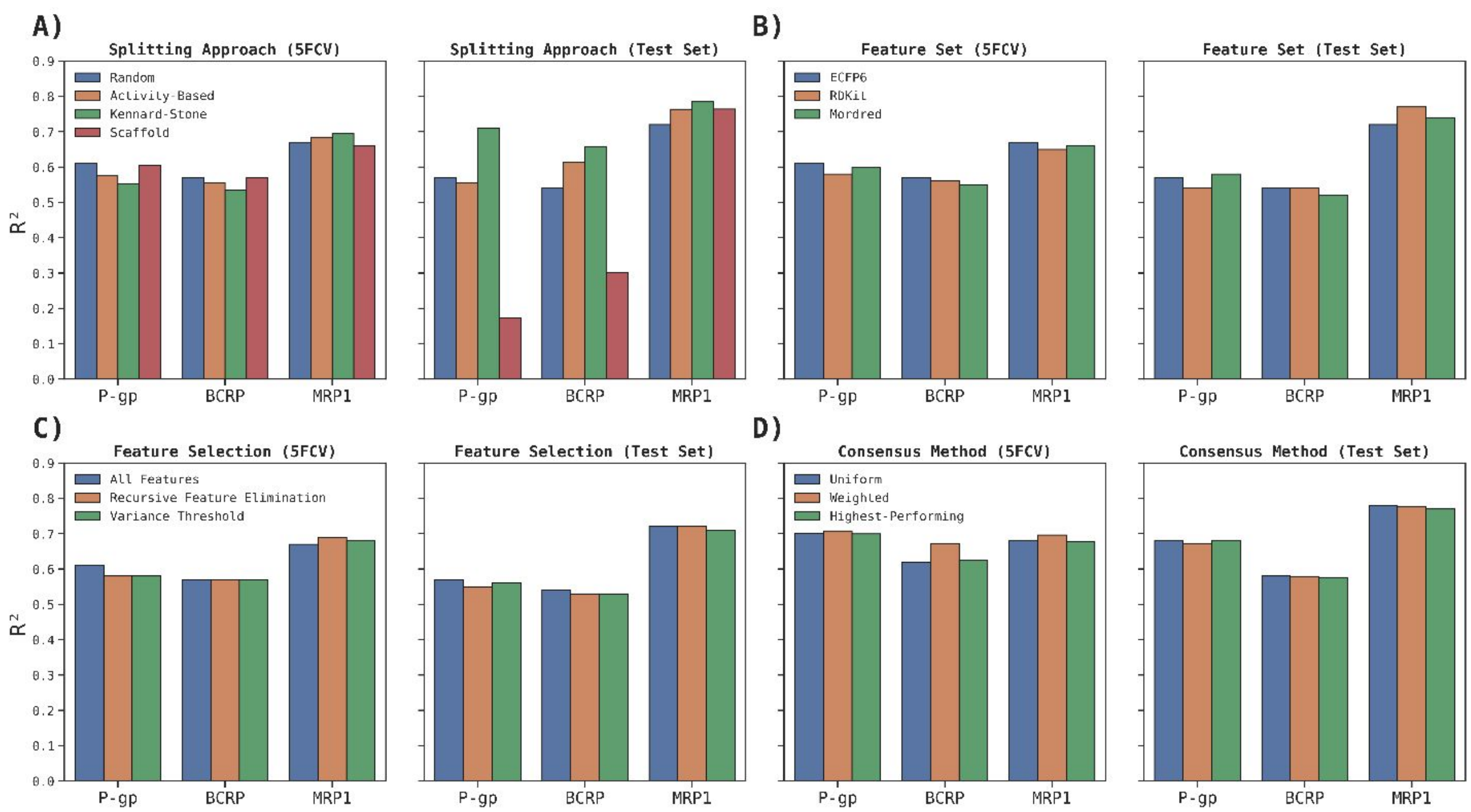

Figure S4. Comparison of model performances (R^2) using various **(A)** data splitting approaches, **(B)** chemical descriptor sets, **(C)** feature selection methods, and **(D)** consensus modeling methods.

Table S4. Individual and consensus model mean absolute error performances.

Algorithm	Descriptor	Mean Absolute Error (MAE; Mean ± Standard Deviation)					
		**P-gp**		**BCRP**		**MRP1**	
		**5FCV**	**Test**	**5FCV**	**Test**	**5FCV**	**Test**
RF	ECFP6	0.46 ± 0.04	0.48	0.39 ± 0.02	0.40	**0.37 ± 0.04**	0.37
	FCFP6	0.47 ± 0.04	0.47	0.41 ± 0.01	0.41	0.38 ± 0.03	0.36
	MACCS	0.45 ± 0.03	0.42	0.42 ± 0.02	0.42	0.41 ± 0.02	**0.32**
	RDKit	0.47 ± 0.03	0.47	0.40 ± 0.02	0.40	0.38 ± 0.03	**0.32**
SVM	ECFP6	0.43 ± 0.02	0.45	0.40 ± 0.03	0.41	0.41 ± 0.06	0.44
	FCFP6	0.43 ± 0.01	0.44	0.39 ± 0.03	0.40	0.42 ± 0.06	0.38
	MACCS	0.45 ± 0.03	0.41	0.43 ± 0.04	0.39	0.41 ± 0.03	0.34
	RDKit	0.43 ± 0.02	**0.38**	0.43 ± 0.02	0.41	0.38 ± 0.04	0.35
XGB	ECFP6	0.42 ± 0.03	0.44	0.40 ± 0.01	0.42	0.40 ± 0.05	0.39
	FCFP6	0.43 ± 0.02	0.44	0.39 ± 0.02	0.41	0.40 ± 0.04	0.38
	MACCS	0.46 ± 0.03	0.42	0.44 ± 0.03	0.46	0.44 ± 0.03	0.35
	RDKit	0.45 ± 0.02	0.43	0.42 ± 0.02	0.41	0.40 ± 0.05	0.33
*k*NN	ECFP6	0.44 ± 0.02	0.45	0.43 ± 0.01	0.43	0.40 ± 0.06	0.35
	FCFP6	0.45 ± 0.02	0.45	0.44 ± 0.02	0.42	0.44 ± 0.07	0.42
	MACCS	0.45 ± 0.02	0.41	0.48 ± 0.04	0.43	0.43 ± 0.04	0.35
	RDKit	0.44 ± 0.03	0.40	0.46 ± 0.02	0.46	0.41 ± 0.03	0.37
GCN	Graphs	0.49 ± 0.03	0.47	0.44 ± 0.02	0.40	0.48 ± 0.06	0.37
Consensus	-------------	**0.40 ± 0.03**	0.39	**0.38 ± 0.02**	**0.38**	**0.37 ± 0.04**	**0.32**

*Best performing five-fold cross validation (5FCV) and test set model MAE for each transporter are shown in bold.

Table S5. Individual and consensus model root mean squared error performances.

**Algorithm**	**Descriptor**	**Root Mean Squared Error (RMSE; Mean ± Standard Deviation)**					
		**P-gp**		**BCRP**		**MRP1**	
		**5FCV**	**Test**	**5FCV**	**Test**	**5FCV**	**Test**
RF	ECFP6	0.60 ± 0.06	0.60	0.53 ± 0.03	0.53	0.51 ± 0.09	0.46
	FCFP6	0.63 ± 0.05	0.59	0.54 ± 0.02	0.56	0.52 ± 0.08	0.46
	MACCS	0.60 ± 0.04	0.57	0.56 ± 0.02	0.57	0.57 ± 0.06	0.42
	RDKit	0.63 ± 0.04	0.63	0.54 ± 0.02	0.53	0.52 ± 0.08	0.42
SVM	ECFP6	0.57 ± 0.04	0.57	0.54 ± 0.03	0.54	0.54 ± 0.09	0.56
	FCFP6	0.58 ± 0.02	0.59	0.52 ± 0.03	0.53	0.55 ± 0.10	0.47
	MACCS	0.61 ± 0.04	0.58	0.57 ± 0.04	0.53	0.55 ± 0.08	0.49
	RDKit	0.60 ± 0.02	0.55	0.57 ± 0.02	0.55	0.52 ± 0.08	0.42
XGB	ECFP6	0.58 ± 0.04	0.59	0.54 ± 0.03	0.58	0.55 ± 0.09	0.51
	FCFP6	0.60 ± 0.03	0.60	0.53 ± 0.03	0.57	0.54 ± 0.10	0.51
	MACCS	0.62 ± 0.04	0.60	0.59 ± 0.03	0.64	0.63 ± 0.08	0.46
	RDKit	0.61 ± 0.02	0.58	0.57 ± 0.03	0.55	0.55 ± 0.11	**0.41**
*k*NN	ECFP6	0.61 ± 0.03	0.63	0.59 ± 0.02	0.57	0.54 ± 0.07	0.48
	FCFP6	0.62 ± 0.03	0.64	0.60 ± 0.03	0.57	0.59 ± 0.10	0.57
	MACCS	0.63 ± 0.06	0.59	0.64 ± 0.04	0.56	0.60 ± 0.10	0.44
	RDKit	0.61 ± 0.03	0.56	0.61 ± 0.02	0.61	0.56 ± 0.08	0.46
GCN	Graphs	0.65 ± 0.04	0.63	0.58 ± 0.02	0.55	0.63 ± 0.07	0.48
Consensus	-------------	**0.53 ± 0.03**	**0.52**	**0.50 ± 0.03**	**0.51**	**0.50 ± 0.08**	**0.41**

*Best performing five-fold cross validation (5FCV) and test set model RMSE for each transporter are shown in bold.

Table S6. Comparison of activities of eight retested training set compounds using higher concentrations of test chemical up to 100 μM in transporter-overexpressing cells.

Compound	Transporter	New Experimental IC_{50} (μM)[a]	Training Set IC_{50} (μM)[b]
Tariquidar	P-gp	0.043	0.105
Encequidar	P-gp	1.090	0.009
Combretastatin A4	P-gp	N/A (non-inhibitor)	0.009
ONT-093	P-gp	1.705	0.032
Avapritinib	BCRP	3.000	0.002
Febuxostat	BCRP	15.47	0.027
Ponatinib	BCRP	0.200	0.040
Tivozanib	BCRP	1.195	0.069

[a] Half-maximal inhibitory concentration (IC_{50}) measured in transporter-overexpressing cells.
[b] Half-maximal inhibitory concentration (IC_{50}) from curated dataset.

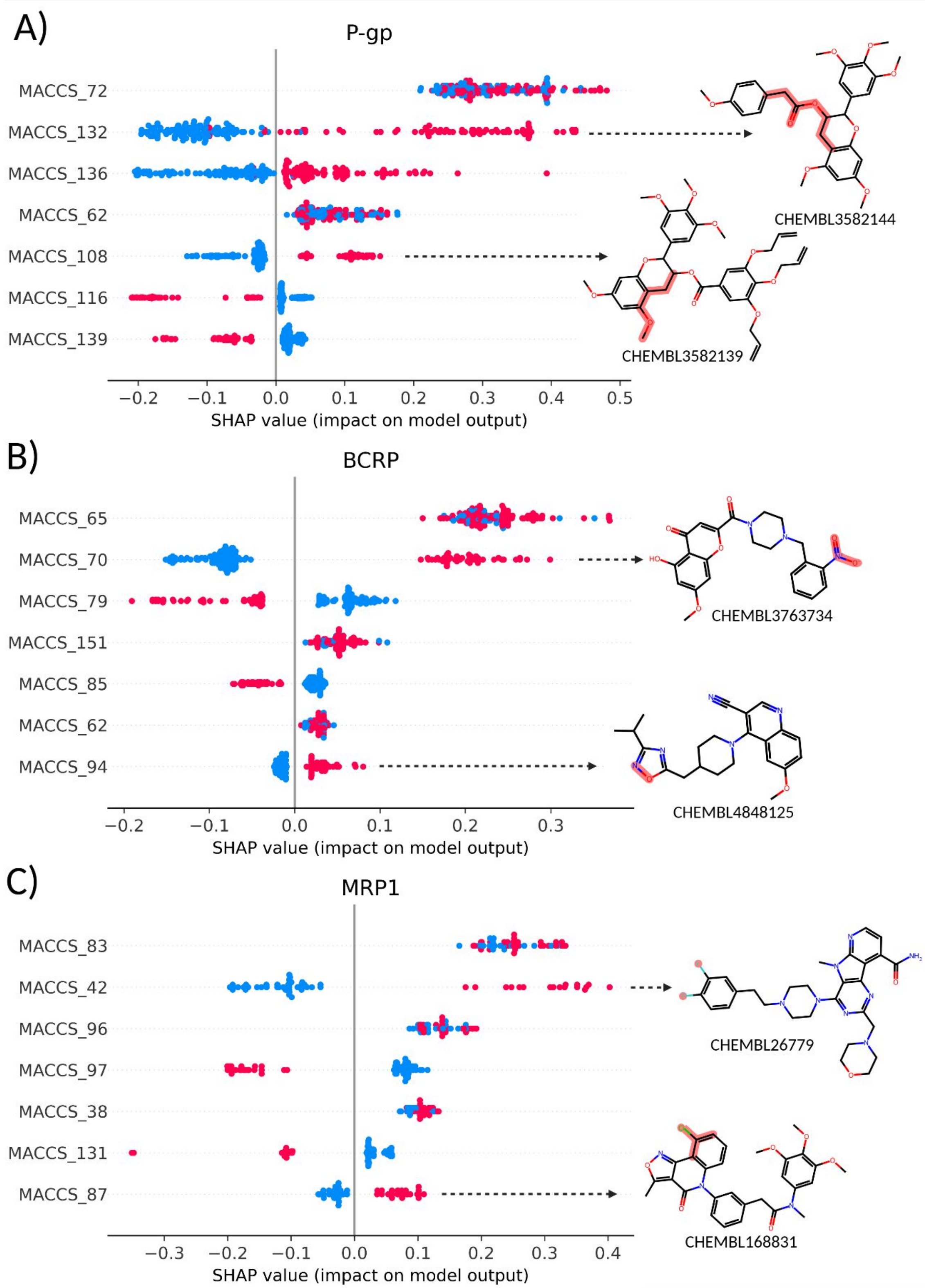

Figure S5. Top MACCS descriptors in the **(A)** P-gp, **(B)** BCRP, and **(C)** MRP1 random forest models. Representative compounds for the most differentiated top MACCS descriptors shown with highlighted substructures. Red points represent the presence of a chemical substructure, where blue points represent the absence of a chemical substructure.

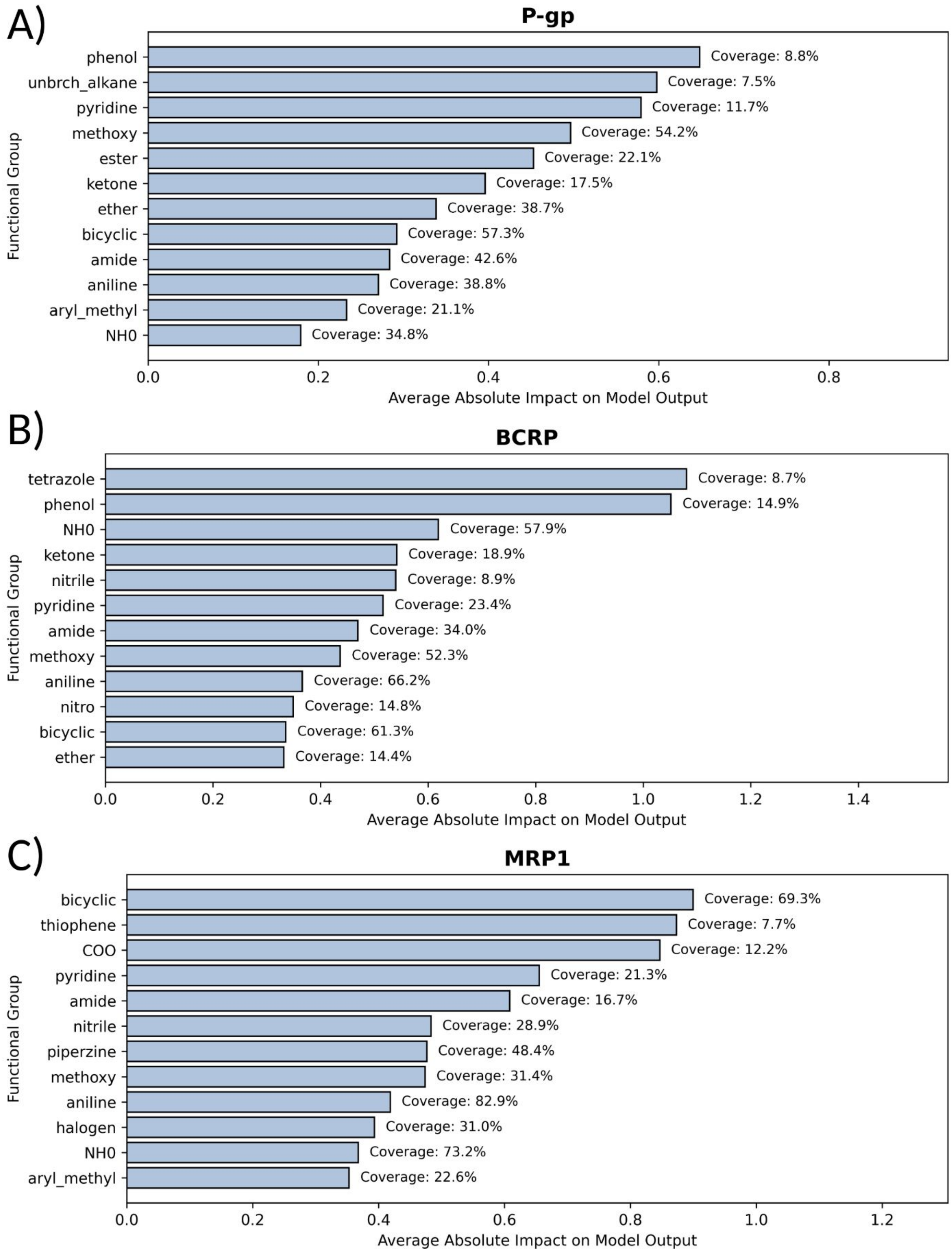

Figure S6. Top global contributing functional groups identified through local interpretable model-agnostic explanations (LIME) analysis. Coverage represents the percentage of compounds in the dataset which contain the functional group.
